# Altered generation of ciliated cells in chronic obstructive pulmonary disease

**DOI:** 10.1038/s41598-019-54292-x

**Published:** 2019-11-29

**Authors:** Sophie Gohy, François M. Carlier, Chantal Fregimilicka, Bruno Detry, Marylène Lecocq, Maha Zohra Ladjemi, Stijn Verleden, Delphine Hoton, Birgit Weynand, Caroline Bouzin, Charles Pilette

**Affiliations:** 1 Université catholique de Louvain (UCL), Institute of Experimental & Clinical Research - Pole of Pneumology, ENT and Dermatology, Avenue Hippocrate 54/B1-54.04, B-1200 Brussels, Belgium; 2 Department of Pneumology, Cliniques universitaires Saint-Luc, Avenue Hippocrate 10, B-1200 Brussels, Belgium; 3 Université catholique de Louvain (UCL), Institute of Experimental & Clinical Research - IREC Imaging Platform, Avenue Hippocrate 55/B1-55.20, B-1200 Brussels, Belgium; 4 Department of Pneumology, UZ Leuven, Herestraat 49, B-3000 Leuven, Belgium; 5 Department of Pathology, Cliniques universitaires Saint-Luc, Avenue Hippocrate 10, B-1200 Brussels, Belgium; 6 Department of Pathology, UZ Leuven, Herestraat 49, B-3000 Leuven, Belgium

**Keywords:** Mechanisms of disease, Chronic obstructive pulmonary disease

## Abstract

In COPD, epithelial changes are prominent features in the airways, such as goblet cell hyperplasia and squamous metaplasia. In contrast, it remains unclear whether ciliated cells are reduced and which pathways dysregulate epithelial differentiation. We hypothesized that bronchial epithelial cell lineage specification is dysregulated in COPD because of an aberrant reprogramming through transforming growth factor (TGF)-β1. Surgical lung tissue from 81 COPD and 61 control (smokers and non-smokers) patients was assessed for bronchial epithelial cell phenotyping by immunohistochemistry, both *in situ* and *in vitro* in reconstituted air-liquid interface (ALI) cultures. The role of TGF-β1 was studied *in vitro*. COPD epithelium in large airways, when compared to controls, showed decreased β-tubulin IV + ciliated cells (4.4%, 2.5–8.8% versus 8.5%, 6.3–11.8% of surface staining, median and IQR, p = 0.0009) and increased MUC5AC + goblet cells (34.8%, 24.4–41.9% versus 10.3%, 5.1–17.6%, p < 0.0001). Both features were recapitulated in the ALI-cultured epithelium from COPD patients. Exogenous TGF-β1 reduced mucociliary differentiation while neutralizing TGF-β1 during ALI increased both specialized cell types. The COPD airway epithelium displays altered differentiation for ciliated cells, which recapitulates *in vitro*, at least in part through TGF-β1.

## Introduction

Chronic obstructive pulmonary disease (COPD) is a frequent and major respiratory disease, characterized by a progressive and poorly reversible airflow limitation in subjects exposed to noxious particles and gases, most frequently from cigarette smoke. The disease is associated with an abnormal chronic inflammatory response of the airways^1^, with activation of neutrophils and macrophages and imbalance between proteinases/anti-proteinases and oxidants/anti-oxidants^2^. In addition, structural changes in COPD airways occur and involve dysregulation of the epithelial-mesenchymal unit, with epithelial modifications and subepithelial fibrosis^3^.

The airways are covered by a muco-ciliated, pseudostratified epithelium, consisting of four main cell types^4^, namely ciliated cells (at least 50%, possessing more than 300 cilia per cell allowing mucus clearance), mucus-producing goblet cells (5 to 15% in proximal airways, virtually absent in distal airways)^5^, basal cells (acting as progenitor cells), and CC16-producing club (ex Clara) cells^6^. Mucociliary clearance represents a fundamental defence mechanism that requires the cooperation between goblet and ciliated cells. Ciliated cell commitment is initially driven through activation of Notch signaling, followed by the upregulation of hundreds of structural and regulatory ciliary genes of the multiciliated cell program. Among the transcriptional network involved in ciliogenesis, Forkhead/winged helix proteins (notably FOXJ1) have a central role by regulating more than 500 genes^7^. Motile cilium biogenesis involves the replication, trafficking and apical docking of basal bodies in the plasma membrane followed by the elongation of axonemes^8^. The axoneme of motile cilia, the cylindrical scaffold, contains hundreds of proteins organized in nine peripheral microtubules doublets (consisting of α- and β-tubules), one central pair and dynein arms. Recently, Perotin *et al*. showed that primary cilia (non-motile) are dysregulated in COPD patients both in remodelled and non-remodelled epithelium^9^. More broadly, ciliopathies are particularly prone to induce cystic diseases at various sites, notably the lung in primary ciliary dyskinesia (bronchiectasis) or the kidneys (in polycystic kidney disease)^10,11^.

Several changes of the bronchial epithelium have been reported in COPD. Basal cell hyperplasia is an early abnormality described in smokers and COPD that leads to squamous metaplasia or goblet cell hyperplasia^12^. An important hallmark of the disease is the excess of mucus, due to increases in goblet cells and in mucus secretion^13,14^ which have been directly related to cigarette smoke exposure^15^ and to the activation by inflammatory mediators such as interleukin (IL)-4, IL-13^16^, IL-17^17^, tumour necrosis factor, neutrophil elastase^18^ and S100 proteins^19^. In contrast, a concomitant loss in ciliated cells is less well documented in COPD^3^, while reduced beat frequency^20^, changes in the nasal mucosa^21^ as well as smoke-induced loss^22^ or shortening^23,24^ of the cilia have been reported. Besides IL-13, which may promote goblet cell hyperplasia and loss of cilia *in vitro*^25^ with relevance to asthma, transforming growth factor (TGF)-β is upregulated in the bronchial epithelium and in air-liquid interface (ALI)-human bronchial epithelial cells (HEBC) from COPD patients^26^ and has a central role in the remodeling of the epithelium in this disease^27^.

The present study was therefore designed to directly assess bronchial epithelial cell differentiation both in lung tissue and bronchial epithelial cultures differentiated upon ALI, to address the hypothesis that ciliated cell numbers are reduced in conducting airways from COPD and that this feature reflects an epithelial dysregulation that could persist *ex vivo* as a result of an aberrant reprogramming through TGF-β1.

## Results

### Altered bronchial epithelial differentiation in COPD

**Figure 1:**
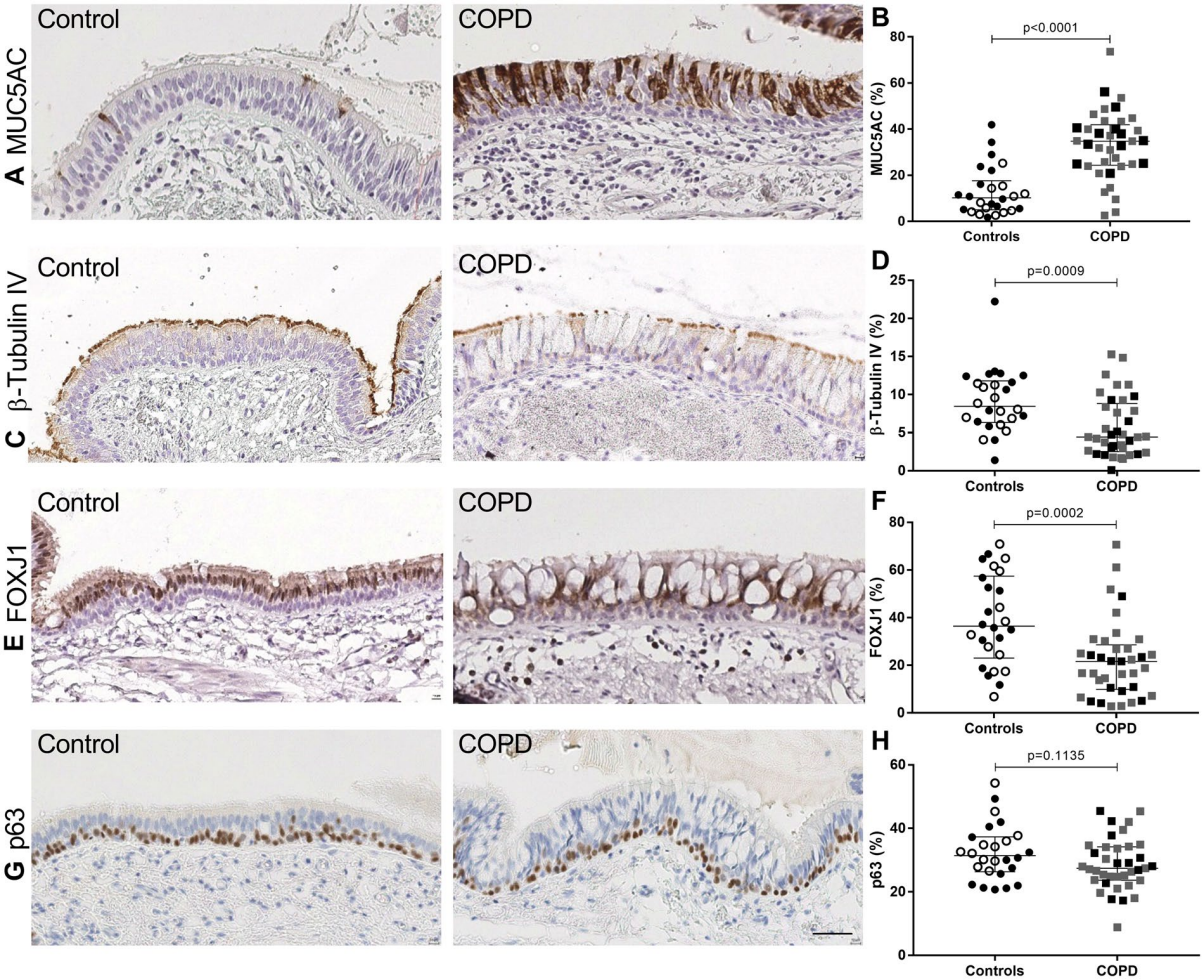
Cell lineage immunophenotyping in large airways. (**A**) IHC for MUC5AC (goblet cells), in large airways of a control and a COPD patient. (**B)** Quantification of MUC5AC staining in large airways expressed in percentage of positive area (n = 63). (**C**) IHC for ß-tubulin IV (ciliated cells) in large airways of a control and a COPD patient. (**D**) Quantification of ß-tubulin IV staining in large airways expressed in percentage of positive area (n = 63). (**E**) IHC for FOXJ1 staining (ciliated cells) in large airways of a control and a COPD patient. (**F**) Quantification of FOXJ1 staining in large airways expressed in percentage of positive cells (n = 63). (**G)** IHC for p63 (basal cells) in large airways of a control and a COPD patient. (**H**) Quantification of p63 staining in large airways expressed in percentage of positive cells (n = 63). Scale bar, 50 µm. White dots represent non-smoker controls and black dots current smoker controls, grey squares represent mild and moderate COPD and black squares severe and very severe COPD. Mann-Whitney U test.

**Figure 2:**
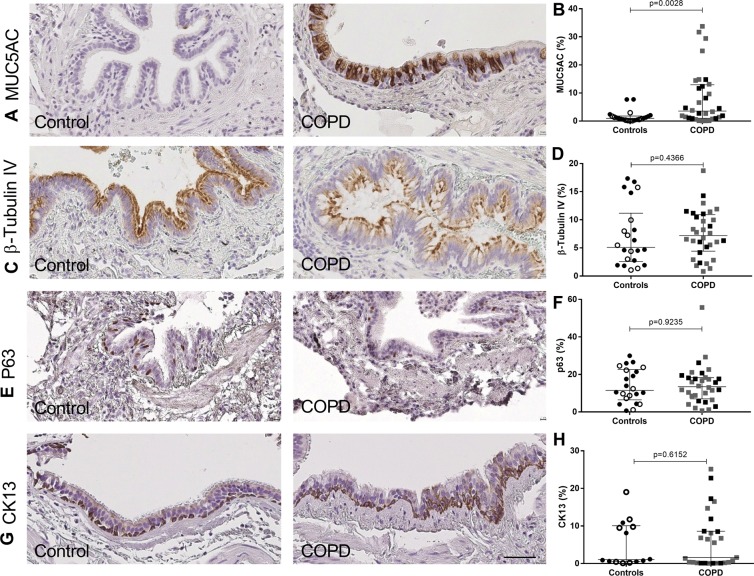
Cell lineage immunophenotyping in small airways. (**A**) IHC for MUC5AC (goblet cells), in small airways of a control and a COPD patient. (**B)** Quantification of MUC5AC staining in small airways expressed in percentage of positive area (n = 54). (**C)** IHC for ß-tubulin IV (ciliated cells) in small airways of a control and a COPD patient. (**D)** Quantification of ß-tubulin IV staining in small airways expressed in percentage of positive area (n = 54). (**E**) IHC for p63 (basal cells) in small airways of a control and a COPD patient. (**F)** Quantification of p63 staining in small airways expressed in percentage of positive cells (n = 54). (**G**) IHC for CK13 (basal cells) in small airways of a control and a COPD patient. (**H**) Quantification of CK13 staining in small airways expressed in percentage of positive cells (n = 41). Scale bar, 50 µm. White dots represent non-smoker controls and black dots current smoker controls, grey squares represent mild and moderate COPD and black squares severe and very severe COPD. Mann-Whitney U test.

In our study population (Table 1), we first addressed whether proportions of goblet, ciliated and basal cells were modified in conducting airways of COPD patients, immunostained for bronchial epithelial lineage markers. In the large and small airway epithelium from COPD patients, MUC5AC (one of the main glycoproteins of the mucus) expression was increased in COPD compared to controls (Figs. 1, 2A,B and E1). In contrast, β-tubulin IV + (forming with α-tubulin, the structural subunit of the microtubules) and FOXJ1 + (main transcription factor of ciliary differentiation) ciliated cells were decreased in COPD compared to controls in large airways (Figs. 1C–F, E1), whereas p63 + basal cells were not affected (Fig. 1G,H). Changes in MUC5AC expression in large and small airways were linked to tobacco status as active smokers displayed increased MUC5AC expression compared to non-smokers (Fig. E2,A,B). No differences in β-tubulin IV + and p63 + /CK13 + basal cells were observed in small airways (Fig. 2C–H). Furthermore, MUC5AC staining in small airways correlated with staining in large airways (Fig. E2,C), and small airway MUC5AC expression correlated in COPD patients with diffusing capacity for carbone monoxide (DL_CO_) (Fig. E2,D). In contrast, β-tubulin IV and FOXJ1 were not correlated to smoking history (data not shown). These data show that goblet cell hyperplasia in COPD is closely related to smoking, whereas the decrease in ciliated cells is specifically observed in COPD.

**Table 1:** Patient characteristics of the study population.

	Non-smokers	(ex)-Smokers	Mild COPD	Moderate COPD	Severe COPD	All
Subjects, n	24	37	24	23	34	142
Sex (F/M)	19/5	16/21	5/19	5/18	15/19	60/82
Smoking history, pack-yrs (n = 127) (never/former/current)	0^†‡§ll^ (24/0/0)	27 ± 17^*ll^ (0/16/21)	37 ± 17^*^ (0/11/13)	42 ± 17^*^ (0/8/15)	45 ± 22^*†^ (0/30/4)	30 ± 23 (24/65/53)
Inhaled corticosteroids, n	1	2	1	4	27	35
Inhaled corticosteroids, BDP equivalent, µg/day (n = 35)	568	1,269 ± 1,418	2,272	1,603 ± 977	1,828 ± 1,989	1,749 ± 1,806
Age, yrs	66 ± 12	62 ± 14	65 ± 10	65 ± 10	60 ± 5	63 ± 11
BMI, kg/m²	26.1^ll^ ± 3.9	25 ± 5.4	25.9 ± 5.8^ll^	24.3 ± 4.3	21.9 ± 4.4^‡*^	24.5 ± 5.0
FEV1, % predicted (n = 136)	93 ± 16^§ll^	97 ± 21^§ll^	94 ± 15^§ll^	68 ± 8^*†‡ll^	27 ± 10^*†‡§^	75 ± 32
FEV1/FVC ratio, % (n = 135)	78 ± 7^‡§ll^	78 ± 7^‡§ll^	65 ± 3^*†ll^	60 ± 9^*†ll^	36 ± 12^*†‡§^	63 ± 19
DL_CO_, % predicted (n = 106)	81 ± 18^§ll^	75 ± 14^§ll^	72 ± 21^ll^	58 ± 17^*†‡^	34 ± 15^*†‡§^	65 ± 23
Lung tissue (IHC in LA, Fig. 1)	12	14	12	13	12	63
Lung tissue (IHC in SA, Fig. 2)	9	13	10	11	11	54
HBEC (IHC, Fig. 3)	8	13	5	7	7	40
HBEC (WB, Fig. 3)	3	7	4	2	10	26
HBEC (Whole-mount, Fig. 4)	3	2	0	0	5	10
HBEC (RTqPCR, Fig. 5)	5	7	11	6	10	39

Demographic data, lung function tests, smoking history and inhaled corticotherapy are stated for the patient groups, classified according to smoking history and the presence of airflow limitation. N is specified when data are missing. Data are means ± SD.

*Definition of abbreviations*: F, female; M, male; yrs, years; BDP, beclomethasone diproprionate. BMI, body mass index; FEV1, forced expiratory volume in one second; FVC, forced vital capacity; DL_CO_, diffusing capacity of the lung for carbon monoxide; IHC, immunohistochemistry; LA, large airways; SA, small airways; WB, western blot; RTqPCR, real-time quantitative polymerase chain reaction.

^*^p < 0.05 versus non-smokers, ^†^p < 0.05 versus (ex)-smokers, ^‡^p < 0.05 versus mild COPD, ^§^p < 0.05 versus moderate COPD, ll p < 0.05 versus severe COPD.

### Altered mucociliary differentiation in the cultured COPD epithelium

**Figure 3:**
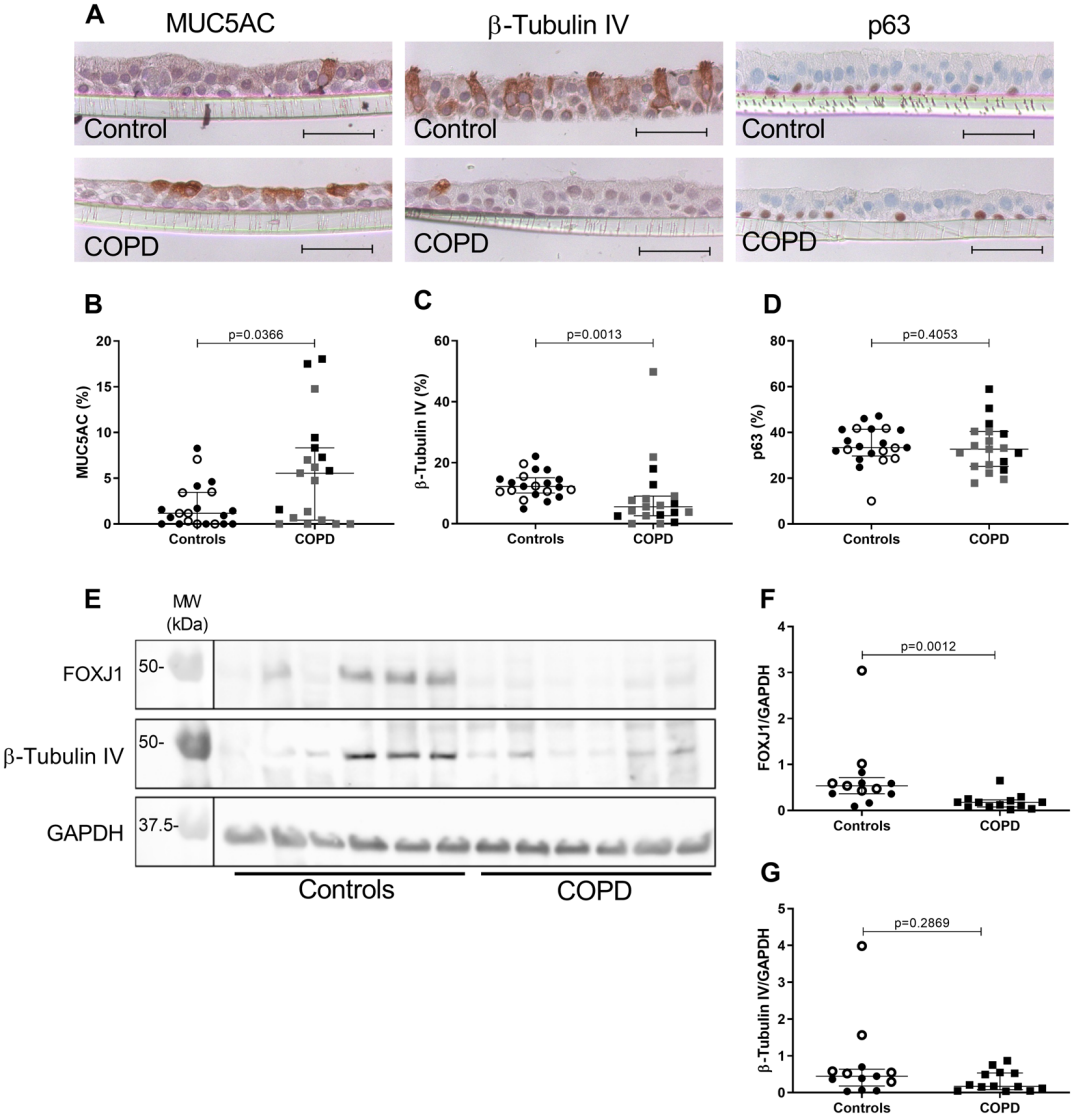
Cell lineage immunophenotyping in ALI-HBEC. (**A)** IHC for MUC5AC (goblet cells), ß-tubulin IV (ciliated cells) and p63 (basal cells) in ALI-HBEC of a control and a COPD patient. (**B**) Quantification of MUC5AC staining in ALI-HBEC expressed in percentage of positive cells (n = 40). (**C**) Quantification of ß-tubulin IV staining in ALI-HBEC expressed in percentage of positive cells (n = 40). (**D**) Quantification of p63 staining in ALI-HBEC expressed in percentage of positive cells (n = 40). (**E)** Immunoblot for FOXJ1 (50 kDa), ß-tubulin IV (50 kDa) and GAPDH (37 kDa) expression in cellular lysates of ALI-HBEC from controls and COPD patients (n = 12). (**F**) Quantification of FOXJ1 protein, as referred to GAPDH in cellular lysates of ALI-HBEC from controls and COPD patients (n = 26). (**G**) Quantification of ß-tubulin IV protein, as referred to GAPDH in cellular lysates of ALI-HBEC from controls and COPD patients (n = 26). Scale bar, 50 µm. White dots represent non-smoker controls and black dots current smoker controls, grey squares represent mild and moderate COPD and black squares severe and very severe COPD. Mann-Whitney U test.

**Figure 4:**
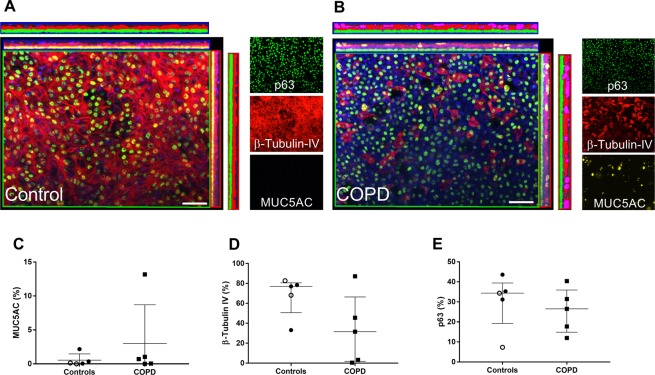
Cell lineage immunophenotyping in ALI-HBEC by immunofluorescence. (**A,B)** Immunofluorescence for MUC5AC (yellow staining and pink quantification mask), ß-tubulin IV (red staining and quantification mask) and p63 (green staining and quantification mask) in ALI-HBEC from one control non-smoker (**A**) and one severe COPD patient (**B**). Nucleus are stained in blue by DAPI. (**C**) Quantification of MUC5AC staining in ALI-HBEC expressed in percentage of positive area (n = 10). (**D**) Quantification of ß-tubulin IV staining in ALI-HBEC expressed in percentage of positive area (n = 10). (**E**) Quantification of p63 staining in ALI-HBEC expressed in percentage of positive area (n = 10). Scale bar, 50 µm. White dots represent non-smoker controls and black dots current smoker controls and black squares represent severe and very severe COPD.

To evaluate the cellular lineages and their regulation in the respiratory epithelium, we used ALI cultures as an *in vitro* model to study the differentiation process. We found that the bronchial epithelium reconstituted from large airway tissue of COPD patients cultured upon ALI for 2 weeks, recapitulated the epithelial features observed *in situ*, namely increased MUC5AC and decreased β-tubulin IV expression with no change in p63 expression (Fig. 3A–D). In addition, MUC5AC expression in COPD patients correlated negatively with airway obstruction in terms of FEV1 and FEV1/FVC ratio (Fig. E3,A,B) (while β-tubulin IV in current and former smokers negatively correlated with pack years (Fig. E3,C). We also found a correlation between MUC5AC expression in ALI-HBEC and in large airways epithelium (Fig. E2,D). The defect in ciliated cells in COPD was confirmed by a decreased expression of FOXJ1 and β-tubulin IV assessed by western blot (Fig. 3E–G) and by whole-mount immunostaining of 5 controls and 5 COPD patients (Fig. 4A–E). No increased death cell was observed between control and COPD cultures (Supplemental Data, Table E1)

**Figure 5:**
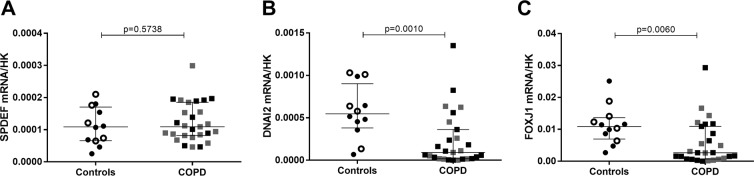
Mucociliary differentiation transcription factors expression in ALI-HBEC. (**A**) SPDEF mRNA expression by RT-qPCR in ALI-HBEC from control and COPD patients, normalized to the geometric mean of the three housekeeping genes (n = 39). (**B)** DNAI2 mRNA expression by RT-qPCR in ALI-HBEC from control and COPD patients, normalized to the geometric mean of the three housekeeping genes (n = 39). (**C**) FOXJ1 mRNA expression by RT-qPCR in ALI-HBEC from control and COPD patients, normalized to the geometric mean of the three housekeeping genes (n = 39). White dots represent non-smoker controls and black dots current smoker controls and black squares represent severe and very severe COPD. Mann-Whitney U test.

To further confirm a transcriptional altered programming of COPD bronchial epithelial cells suggested by the decrease of FOXJ1 protein by immunohistochemistry (IHC) and western blot (WB), transcription factors involved in differentiation of goblet cells (SPDEF) and ciliated cells (FOXJ1) were assessed by real time quantitative polymerase chain reaction (RT-qPCR). Axonemal dynein intermediate chain 2 (DNAI2, a part of the dynein arm of the cilia) messenger ribonucleic acid (mRNA), was measured to support the decrease in structural component of the cilia (suggested by the β-tubulin IV decrease in IHC and WB). No difference was found in SPDEF mRNA expression between COPD and control-derived HBEC either suggesting post-transcriptional regulation or the implication of other pathways controlling goblet cell differentiation, survival and/or proliferation in COPD. In contrast, DNAI2 and FOXJ1 mRNA were both decreased in HBEC cultures from COPD patients as compared to those from controls (Fig. 5A–C). DNAI2 mRNA in current and former smokers correlated with smoking history (Fig. E4,A). As FOXJ1 is decreased both at transcriptional and translational levels, these data suggest an altered programming of the bronchial epithelial differentiation for ciliated cells in COPD and which persists in the bronchial epithelium reconstituted *ex vivo* from such patients.

### Altered bronchial epithelial differentiation is partly related to TGF-β

**Figure 6:**
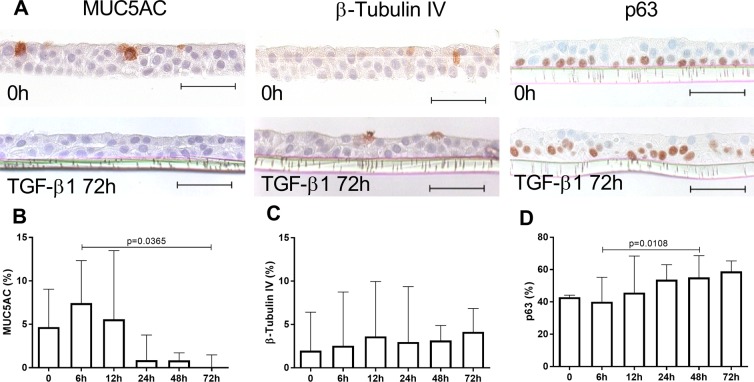
Short-term effect of TGF-β1 on epithelial cell lineages in control ALI-HBEC. (**A**) IHC for MUC5AC (goblet cells), ß-tubulin IV (ciliated cells) and p63 (basal cells) in ALI-HBEC without or with 72 h treatment of TGF-ß1 (10 µg/ml). (**B**) Quantification of MUC5AC staining in ALI-HBEC treated by TGF-ß1 expressed in percentage of positive cells (n = 4). (**C**) Quantification of ß-tubulin IV staining in ALI-HBEC treated by TGF-ß1 expressed in percentage of positive cells (n = 4). (**D**) Quantification of p63 staining in ALI-HBEC treated by TGF-ß1 expressed in percentage of positive cells (n = 5). Scale bar, 50 µm. Friedman test and Dunn’s multiple comparison test.

**Figure 7:**
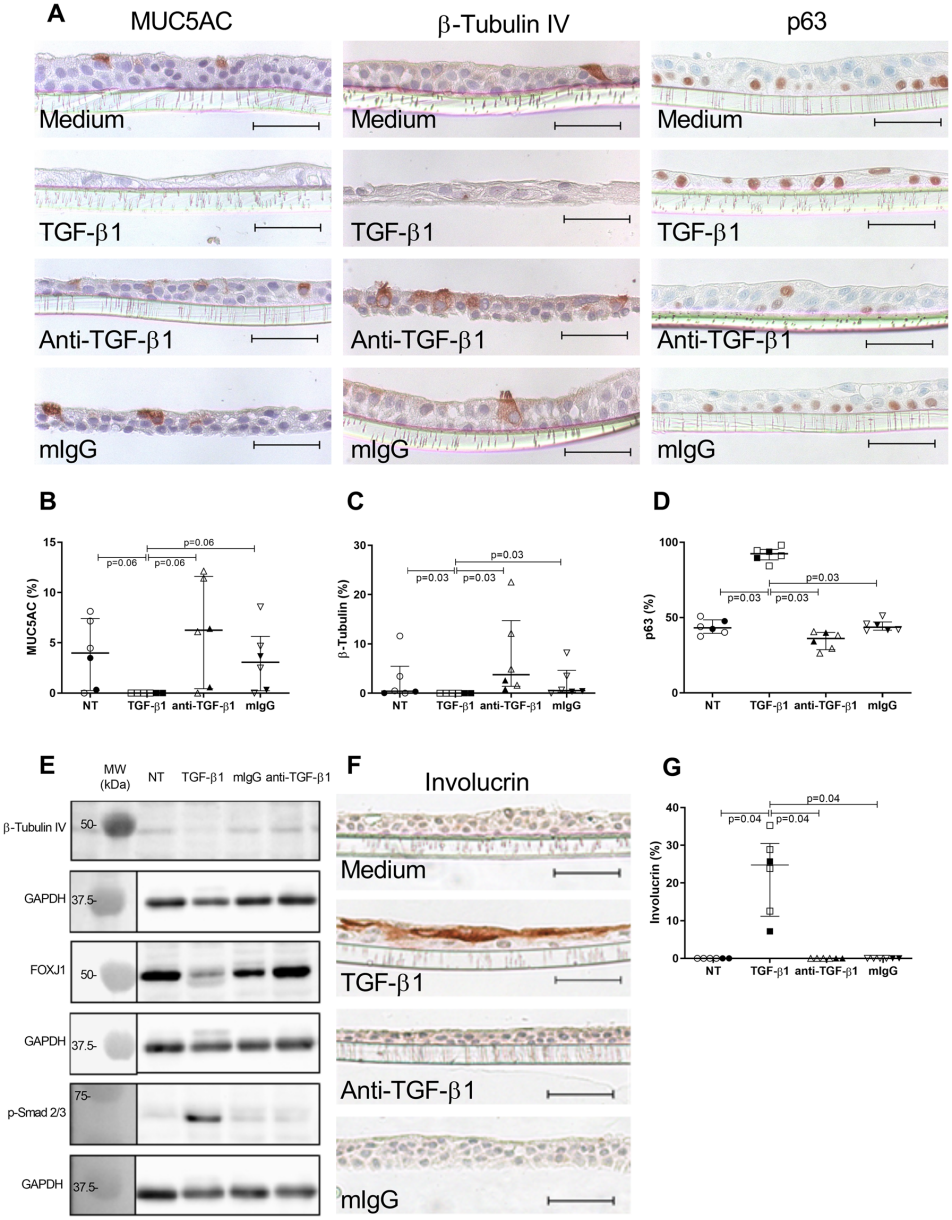
Long-term effect of TGF-β1 and anti-TGF-β1 antibody on epithelial cell lineages. (**A**) IHC for MUC5AC (goblet cells), ß-tubulin IV (ciliated cells) and p63 (basal cells) in ALI-HBEC using TGF-β1 (10 ng/ml), anti-TGF-β1 antibody (10 µg/ml) and control mouse IgG (10 µg/ml) during the 2 weeks of ALI differentiation (pictures are from a control ex-smoker). (**B**) Quantification of MUC5AC staining expressed in percentage of positive cells (n = 6), including 4 controls and 2 COPD donors as white and black dots, respectively). (**C**) Quantification of ß-tubulin IV staining expressed in percentage of positive cells (n = 6, including 4 controls and 2 COPD donors as white and black dots, respectively). (**D**) Quantification of p63 staining expressed in percentage of positive cells (n = 6, including 4 controls and 2 COPD donors as white and black dots, respectively). (**E**) Immunoblot for FOXJ1 (50 kDa), ß-tubulin IV (50 kDa), phospho-Smad2/3 (68 kDa) and GAPDH (37 kDa) expression in cellular lysates of ALI-HBEC from controls and COPD patients (n = 6). (**F**) IHC for involucrin (squamous cells) in ALI-HBEC using TGF-β1 (10 ng/ml), anti-TGF-β1 antibody (10 µg/ml) and control mouse IgG (10 µg/ml) during the 2 weeks of ALI differentiation. (**G)** Quantification of involucrin staining expressed in percentage of positive cells (n = 6, including 4 controls and 2 COPD donors as white and black dots, respectively, pictures are from a control ex-smoker. Scale bar, 50 µm. Friedman test and Dunn’s multiple comparison test.

TGF-β1 was evaluated as a candidate cytokine for dysregulating bronchial epithelial differentiation in COPD as we previously showed that TGF-β1 expression is increased both in bronchial epithelium of large airways and in ALI-HBEC from COPD patients^26^. First, in kinetic experiments on controls HBEC, exogenous TGF-β1 started to decrease MUC5AC+ cells from 24 h and reached significance at 72 hours of treatment (Fig. 6A,B). There was no significant effect on β-tubulin IV+ ciliated cells after 72 hours (Fig. 6A,C) whereas p63+ basal cells slightly increased concomitantly to the decrease in goblet cells (Fig. 6A,D). When treatment was applied throughout the 2 weeks of ALI differentiation, TGF-β1 profoundly affected the bronchial epithelial morphology, with thin and spindle-shape cells and disappearance of MUC5AC and β-tubulin IV+ cells in favour of p63+ basal cells (Fig. 7A–D). Accordingly, β-tubulin IV and FOXJ1 proteins assayed by western blot were affected by TGF-β1, which was confirmed as activating Smad2/3 phosphorylation (Fig. 7E).

As TGF-β1 is intrinsically upregulated in COPD epithelial cells^26^, the role of this cytokine in driving impairment of differentiation into ciliated cells was evaluated by using a blocking monoclonal antibody in primary cultures (Fig. 7). Treatment of HBEC with anti-TGF-β1 strongly increased β-tubulin IV+ cell numbers (Fig. 7A,C). In parallel, this treatment decreased p63+ cell numbers and tended to increase MUC5AC+ cell numbers (Fig. 7A,B,D). We wondered whether TGF-β1 also induced squamous metaplasia. It was observed that involucrin, a terminal marker of such process, was induced upon TGF-β1 treatment of HBEC (Fig. 7F,G), indicating that this factor not only inhibits mucociliary differentiation but also drives squamous metaplasia.

Altogether, these data show that ciliated cells are decreased in large airways from COPD patients, along with increased goblet cells that are also present in their small airways. TGF-β1 is probably involved in this impaired generation of ciliated cells, whereas goblet cell hyperplasia relates to other, TGF-β-independent, mechanisms.

## Discussion

This study shows that the COPD bronchial epithelium is imprinted by an altered programming of lineage differentiation that includes a defect in the generation of ciliated cells that is seen in large conducting airways and recapitulates *ex vivo* following reconstitution upon ALI culture. In addition, this defect is paralleled by an increased differentiation in goblet cells, mainly related to smoking history. This study also provides evidence that TGF-β1 is involved in the defect of ciliated cell differentiation, as well as in squamous metaplasia, while goblet cell hyperplasia occurs through different mechanisms.

Remodeling of the respiratory epithelium is a change in mass, size or composition of the tissue component that represents a hallmark feature of most respiratory diseases. In COPD, it includes goblet cell hyperplasia, squamous metaplasia, basal cell hyperplasia as well as cilia shortening and altered barrier integrity^3,14,28^. In asthma and cystic fibrosis, goblet cell hyperplasia is a prominent feature. In murine experimental asthma model, it has been reported that the number of ciliated cells tended to decrease, but less clearly than club/Clara cells^29^ while the loss of cilia has been reported following smoke exposure^22^ as well as in cystic fibrosis^30^.

This study shows that the defect in ciliated cells in COPD is recapitulated *ex vivo*, in the reconstituted bronchial epithelium upon culture in ALI, while a significant correlation between *in situ* (tissue) and *in vitro* data could also be observed for goblet cells (but not for ciliated cells). As this model implies the differentiation of progenitor/stem basal cells, these data could infer that the differentiation programming is intrinsically imprinted in these cells. Besides the possibility that basal cells could display defects in pathways driving selective differentiation towards goblet or ciliated cells, a more global alteration in progenitor competency has been recently showed. Basal cells from smokers and COPD exhibit impaired self-renewal and pluri-potentiality *in vitro*^31^. Other *in vitro* studies showed that basal cells from COPD patients have limited ability to regenerate a fully differentiated epithelium and that DNA methylation alteration could be involved^32^ whereas the success of performing ALI culture up to 28 days could not be related to the underlying respiratory disease but only to the presence of beating cilia in the initial biopsy^33^. An abnormal programming of bronchial epithelial cells has also been shown in COPD for epithelial dedifferentiation through mesenchymal transition^27^, as well as in asthma for goblet cell hyperplasia^34^.

The mechanisms of cilia dysfunction remain unclear. In parallel to basal cell self-renewal dysfunction and pluri-potentiality described by Ghosh^31^, Lam *et al*. implied autophagy pathway through an epigenetic histone deacetylase-6 regulation^35^. The shortening of the cilia and remodeling of the epithelium in smokers and COPD (basal cell hyperplasia, squamous metaplasia) were linked to EGFR activation through EGF and amphiregulin, further highlighting the complexity of the regulation of epithelial differentiation^36^. Previous work also showed that exposure of ALI-HBEC to cigarette smoke extract decreased the number of ciliated cells and increased the number of MUC5AC positive cells after 28 days of exposure^37^. Whereas EGFR activation by EGF mediates squamous metaplasia and EMT^38^, EGFR ligation by amphiregulin induces goblet cell proliferation and to a lesser extent reduced ciliated cell differentiation^36^. Goblet cell hyperplasia may also originate from a transdifferentiation of ciliated cells triggered by a dual signaling through EGFR and IL-13/IL-4Rα^39^. The mechanisms of altered bronchial epithelial programming are thus multifactorial as evidenced in murine and cell culture experimental models. Finally, we did not find the defect in ciliated cells in small airways while goblet cells were increased. This result could be due to technical bias, as the epithelium is thinner, notably in COPD patients, this quantification by IHC could have overestimated the percentage of positive area in COPD patients (giving the tendency of higher proportion of all cell types in COPD small airways). This should be ideally confirmed by a study focusing on small airway tissue and ALI-HBEC originating from the same patients. Alternatively, goblet cell metaplasia in small airways could represent a dominant feature in COPD. TGF-β1 is a central cytokine in chronic airways diseases^40^, notably in COPD through several lines of evidence including a genetic polymorphism^41,42^, increased expression in the COPD airway epithelium^27,43^, and upregulated production in COPD-derived HBEC cultures. Whereas the role of TGF-β1 for tissue fibrosis through fibroblast activation is well known^27,44^, its role in controlling bronchial epithelial differentiation remains controversial. It has been reported that TGF-β1 may induce, *in vitro*, aberrant differentiation of airway epithelial cells through EMT as well as squamous metaplasia, as evidenced by expression of involucrin or transglutaminase^45,46^. Decreased or increased MUC5AC expression through TGF-β2 activation has been described^47,48^, while TGF-β1 had no effect in both studies. In contrast, Lazard *et al*. reported an increase in basal cells and decreases in ciliated and goblet cells upon treatment with TGF-β1 of human nasal epithelial cells in ALI^49^. Conflicting results regarding TGF-β1 could relate to differences in experimental conditions (timing, doses), as well as in the status of the studied epithelium. In murine experimental asthma, intra-peritoneal treatment with anti-TGF-β1 decreased goblet cell numbers and remodeling^50^. In our study, exogenous TGF-β1 affected global mucociliary differentiation and subsequently increased the proportion of uncommitted basal cells, both in control and COPD HBEC, suggesting that the defective generation of ciliated cells in the COPD epithelium could at least partly relate to TGF-β1. In contrast, goblet cell hyperplasia occurs through TGF-β-independent mechanisms, which could consist of pro-inflammatory mediators (such as cytokines or neutrophil elastase) released upon cigarette smoking.

Basal cell hyperplasia is the first abnormality seen at the onset of aberrant bronchial epithelial differentiation in smokers and COPD, probably reflecting impaired progenitor function of these cells that leads to squamous metaplasia or goblet cell hyperplasia^12^. Although it cannot be excluded that some degree of epithelial remodeling underlies this finding, our data indicate that a defect in generating ciliated cells could occur in COPD, independently (at least in part) from obvious squamous metaplasia and that the absence of basal cell hyperplasia in our study could relate to the fact that we excluded area with obvious squamous metaplasia, which clearly contain more p63+ cells. In contrast and as previously reported^13^, enhanced differentiation in goblet cells also occurs in COPD, mainly in relation to smoking history *per se*. It is tempting to speculate that basal cell hyperplasia reflects a global specification defect of these cells to differentiate into ciliated cells, which normally represent the most abundant specialized cell type. Ultimately, it is likely that the defect in ciliated cells and concomitant goblet cell hyperplasia contribute to impaired muco-ciliary clearance in COPD, along with reduced cilia length^23^ and beating frequency^20^.

Some limitations are part of this study. First, lung samples for controls and mild-to-moderate COPD originated from surgical tissue resected for lung cancer whereas those for very severe COPD consisted of lung explants. It remains possible that this bias affected some findings. Similarly, inhaled corticosteroids, although not influencing the success rate of ALI culture^33^, could have partly influenced our observations. Also, a 2-week time-point was selected for analyses of ALI cultures for changes in differentiation to optimize the chance of capturing signals leading to ciliated cells; however, this may have limited the observation of features of terminal differentiation (including ciliogenesis) usually observed at 4 weeks. Finally, activation of cell death that could occur more selectively in ciliated cells following cigarette smoking^37,51,52^ and/or disease progression, could also contribute to our findings, and should be studied in future studies.

Altogether, these data show that the large airway epithelium is imprinted in COPD with a defective differentiation for ciliated cells and recapitulates *ex vivo* at least in part through aberrant activation by TGF-β1 signaling.

## Methods

All experiments were performed in accordance with relevant guidelines and regulations. Additional details are provided in the online Supplementary File.

### Study subjects

One hundred and forty-two patients were enrolled in this study, consisting of 61 controls (24 non-smokers and 37 smokers or ex-smokers) and 81 COPD patients, namely 24 mild (global initiative for chronic obstructive lung disease (GOLD) stage I), 23 moderate (GOLD stage II) and 7 severe COPD (GOLD stage III) undergoing lung resection surgery for a solitary tumor and recruited between 2007 and 2018 (Table 1). Lung explants from 27 very severe COPD (GOLD stage IV) patients were also included. Detailed clinical history was available and patients underwent lung function testing. Patients with other lung diseases were excluded. All patients gave signed informed consent to the study protocol, which was approved by our local ethical committee (Comité d'éthique hospitalo-facultaire des Cliniques universitaires Saint-Luc. Ref. #2007/19MARS/58).

### Lung tissue sampling and processing

Lung sections (containing large airways and small airways) were obtained from surgical specimens and processed for IHC. One additional large airway sample was obtained for primary epithelial cell culture. Among the 142 enrolled patients (Table 1), expression analyses in tissue (IHC) were performed for 63 patients for the large airways and 54 for the small airways and primary bronchial epithelial cultures were derived from 40 patients for IHC, 10 patients for the whole-mount analyses, 39 patients for RTqPCR, 26 for western blot (Table 1).

### Primary cultures of human bronchial epithelial cells

One piece of large, cartilaginous bronchus per patient away from the tumour site was selected to derive HBEC. p63+ cells (basal cells) represent more than 90% of the total cells after culture in submerged conditions. Cultures were carried out in air/liquid interface (ALI) for 2 weeks, to allow re-differentiation into a pseudo-stratified, mucociliary airway epithelium^53^. For *ex vivo* experiments, recombinant human TGF-β1 was added in the basolateral medium (for 6 to 72 hours after 2 weeks of ALI differentiation) in the kinetic experiment. Finally, recombinant TGF-β1, anti-human TGF-β1 antibody or control mouse immunoglobulin (Ig)G were added (every other day with fresh medium) during the 2 weeks of ALI. No significant cytotoxicity was observed (release of lactate dehydrogenase <10%) in the presented conditions or in COPD patients (Table E1).

### Immunoassays for cellular lineage

#### Immunophenotyping for epithelial markers

Serial paraffin sections of lung tissue and of HBEC filters were stained for epithelial lineage markers: p63 for basal cells, FOXJ1 and β-tubulin IV for ciliated cells, MUC5AC for goblets cells and involucrin for squamous metaplasia. Slides were scanned using Leica SCN400 scanner before selecting the areas. Ten well-preserved areas of epithelium of large and small airways (x400 magnification) were manually delineated for each patient for the quantification. Immunostainings were quantified in these areas using TissueIA software. Color deconvolution was applied to each pixel using hematoxylin and DAB matrices of the software. On the DAB matrice, a threshold was adjusted for DAB detection according to intensity (grey values from 0 to 255) on representative stained versus not stained tissue areas. In a similar way, a threshold was also adjusted for tissue detection. These parameters were kept constant throughout the study for each immunostaining. Results were expressed as stained area (below threshold)/tissue area (below threshold) for β-tubulin IV and MUC5AC stainings (highly correlated to cell counts following tissue segmentation^54^) and as percentage of positive cells (positive nuclei/total nuclei) for p63 and FOXJ1 staining. Ciliated cells (FOXJ1 and β-tubulin IV) were quantified outside of areas of obvious remodeling (i.e. with squamous metaplasia, Fig. E1). Of note, however, involucrin immunostaining was only positive in 4 out of the 63 COPD patients (data not shown) and the inclusion of those regions did not change the quantification data presented in Fig. 1.

For HBEC, 10 well-preserved areas of epithelium were taken for each patient for the quantification, using a Zeiss Axiovert 40 microscope and Axiovision software. Quantification of the positive cells was manually done and data were expressed as the percentage of positive cells (positive cells or nuclei/total cells or nuclei) for β-tubulin IV, MUC5AC, p63 and involucrin. Whole-mount β-tubulin IV, MUC5AC, p63 immunofluorescence were quantified as percentage of positive area. Five optical sections for each stained inserts were acquired from the top to the bottom of the inserts by structured illumination using a Zeiss AxioImager equipped with an ApoTome.z1 module (20x Plan-Apochromat objective). From the acquired image stacks, maximum intensity projections were generated for analysis using the image analysis tool Author version 2017.2 (Visiopharm). p63, beta-tubulin-IV and MUC5AC stained pixels were detected using a thresholding classification method. Results were expressed as percentage of stained area.

#### Western blot for epithelial markers and TGF-β signalling

HBEC were assayed for FOXJ1, β-tubulin IV, phospho-Smad 2/3 and GAPDH (for normalization) expression by western blot. ECL Prime chemiluminescent substrate was used to develop the immunochemical signal that was captured by a CCD-camera imager avoiding overexposure. Quantity One software was used for analysis. Each band individually selected was quantitated by densitometry and normalized for the corresponding GAPDH band intensity. All blot are shown in the Supplementary File.

### RT-qPCR analysis for SPDEF, DNAI2, FOXJ1 mRNA

Total RNA was isolated from HBEC and reverse-transcribed. Expression levels of SPDEF, DNAI2, FOXJ1 mRNA were quantified by RT-qPCR and normalized to the geometric mean of three housekeeping genes (glyceraldehyde-3-phosphate dehydrogenase, ribosomal protein S18, RNA18S)^55^.

### Statistical analysis

Results were shown as scatter dot plots with medians and interquartile ranges. All tests used were non-parametric, Mann-Whitney U test (for unpaired data and the analysis of differences between 2 groups) and Friedman test (for paired data) followed by Dunns post hoc test (for multiple comparisons). Correlation coefficients were calculated using Spearman’s rank method. A *p* value less than 0.05 was considered as statistically significant. Statistical analyses were performed using IBM SPSS Statistics (version 24 for Windows, Chicago, USA) and figures were done using GraphPad Prism (version 7.00 for Windows; GraphPad Software, San Diego, USA; www.graphpad.com).

## Supplementary information

Supplementary data
