# Supplementary material for: Altered generation of ciliated cells in chronic obstructive pulmonary disease
Source: Sci Rep. 2019 Nov 29;9:17963. doi: 10.1038/s41598-019-54292-x (PMC6884487; doi:10.1038/s41598-019-54292-x)

Altered generation of ciliated cells in chronic obstructive pulmonary disease

Authors: Sophie Gohy[1,2] MD PhD, François M. Carlier[1] MD, Chantal Fregimilicka[3], Bruno Detry[1], Marylène Lecocq[1], Maha Zohra Ladjemi[1] PhD, Stijn Verleden[4] PhD, Delphine Hoton MD[5], Birgit Weynand[6] MD, Caroline Bouzin[3] PhD and Charles Pilette[1,2] MD PhD.

METHODS

Patients

One hundred and forty-two patients were enrolled in this study, consisting of 61 controls (24 nonsmokers and 37 smokers or ex-smokers) and 81 COPD patients, namely 24 mild (global initiative for chronic obstructive lung disease (GOLD) stage I), 23 moderate (GOLD stage II) and 7 severe COPD (GOLD stage III) undergoing lung resection surgery for a solitary tumor and recruited between 2007 and 2014 (Table 1). Lung explants from 27 very severe COPD (GOLD stage IV) patients were also included. Detailed clinical history was available and patients underwent lung function testing. Ex-smokers had stopped smoking for more than one year. Patients with other lung diseases were excluded. All patients gave signed informed consent to the study protocol, which was approved by our local ethical committee (Comité d'éthique hospitalo-facultaire des Cliniques universitaires Saint-Luc. Ref. #2007/19MARS/58).

Lung tissue sampling and processing

Lung sections were processed for immunochemistry by immediate immersion in 4% formaldehyde in phosphate buffer saline (PBS) at pH 7.4. One large sample was put directly in culture medium for primary epithelial cell culture.

Primary cultures of human bronchial epithelial cells

One piece of large, cartilaginous bronchus per patient away from the tumour site was dissected and submitted to pronase E 1mg/ml (Sigma) digestion in RPMI supplemented with 200U/ml

penicillin and 200µg/ml streptomycin overnight at 4°C in order to derive human broncho-epithelial cells (HBEC). After pronase digestion, cells were cultured in submerged conditions in retinoic acid-supplemented BEGM medium until confluence (± for 10 days). After pronase digestion and after 10 days of submerged culture, cell count was performed on two slides after centrifugation at 900 rpm during 5 min (Shandon Cytospin 3) of cell suspension (in PBS) to reach a total of 50,000 cells per slide. Using cytospin, we analysed epithelial and mesenchymal markers to confirm that p63+ cells (basal cells) represent more than 90% of the total cells after culture in submerged conditions.

Cells were then detached and subcultured on 12-well polyester filter-type inserts (0.4-µm pore size; Corning, New York, USA) coated with 0.2 mg/ml collagen IV (Sigma-Aldrich, Saint-Louis, USA), seeded at a density of 50,000 cells/well until a confluent polarized monolayer is obtained. The culture was then carried out in air/liquid interface (ALI) for 2 weeks, to allow re-differentiation into a pseudo-stratified, mucociliary airway epithelium. ALI-HBEC were cultured in BEBM:DMEM (1:1) medium supplemented with penicillin (100U/ml), streptomycin (100µg/ml), bovine pituitary extract (52µg/ml), insulin (5µg/ml), hydrocortisone (0.5g/ml), transferrin (10µg/ml), epinephrine (0.5µg/ml), Epidermal Growth Factor (0.5ng/ml), triiodothyronine (3.25ng/ml), bovine serum albumin (BSA) (1.5mg/ml) and retinoic acid (30ng/ml) (Lonza, Basel, Switzerland).

For kinetics experiments, recombinant human TGF-β1 (10ng/ml, R&D Systems, Minneapolis, USA) was added in the basolateral compartment during 6 to 72 hours after 2 weeks of ALI differentiation. For blocking experiments, anti-TGF-β1 blocking antibody (10µg/ml, kindly gifted by Pr J. Van Snick, Ludwig Institute, UCL Brussels, Belgium), control mouse IgG (10µg/ml, our lab) or recombinant human TGF-β1 (10ng/ml, R&D Systems, Minneapolis, USA) were added in the basolateral compartment during the 2 weeks of ALI differentiation. At the end of the experiment, basolateral medium was collected and the apical pole of HBEC

was washed twice with 150µl sterile PBS. Apical washes were centrifuged for 5 minutes at 10,000 G and stored at -20°C until assayed. HBEC inserts were kept for IHC (directly immersed in 4% formaldehyde in PBS at pH 7.4 and then embedded in paraffin blocks or used for whole-mount staining). No significant cytotoxicity was observed (release of lactate dehydrogenase < 10%) in the presented conditions (Table E1).

Immunohistochemistry

Serial lung and inserts sections (5 µm thickness) were cut from paraffin blocks, spread on superfrost plus glass slides, and dried at 37°C for at least 24 hours. The slides were then processed for immunohistochemistry, each step of the procedure being followed by washing with tris buffer saline (TBS) with 0.1% Tween 20 (Sigma-Aldrich, Saint-Louis, USA) (pH 7.4) except after blocking step. After deparaffinization, endogenous peroxidases were inhibited by incubation in 0.3% H_2O_2 in water for 30 minutes. Antigen retrieval was performed in a citrate buffer during 75 minutes at 98°C. The slides were treated with 2% (wt/vol) BSA in TBS for 30 minutes and after that for 10 minutes with avidin and biotin 0.001%. Sections were then incubated overnight at 4°C with primary antibody (except for MUC5AC antibody which was incubated for 1 hour at RT), followed by biotinylated antibody (30 minutes, RT) and streptavidin horseradish peroxidase conjugated (HRP) (BD Bioscience, San Jose, USA) (30 minutes, at RT) incubation. Sections were revealed with the Ultra View Universal 3, 3-diaminobenzidine detection kit (Roche, Basel, Switzerland) according to manufacturer's instructions.

Immunostaining was performed during the same run following this protocol using antibodies to p63 (clone 7JUL, Leica, Milton Keyne, UK, 1/30), CK13 (Sigma-Aldrich, Saint-Louis, USA, 13.4µg/ml), β-tubulin IV (clone ONS.1A6, Sigma-Aldrich, Saint-Louis, USA, 1.16µg/ml), human FOXJ1 (polyclonal, Sigma-Aldrich, Saint-Louis, USA, 0.4µg/ml), human MUC5AC (clone CLH2, Leica, Milton Keyne, UK, 1µg/ml), human Involucrin (clone SY5, Thermo Fisher

Scientific, Kent, United Kingdom, 2µg/ml) or mouse IgG as appropriate negative control. Secondary antibodies were biotin-conjugated anti-mouse IgG (whole molecule) (Sigma-Aldrich, Saint-Louis, USA, 1/3000). Slides were scanned using Leica SCN400 scanner (Leica, Wetzlar, Germany) before selecting the areas. Ten well-preserved areas of epithelium of large and small airways (x400 magnification) were manually delineated for each patient for the quantification. Immunostainings were quantified in these areas using TissueIA software (Leica Biosystems, Dublin, Ireland). Color deconvolution was applied to each pixel using hematoxylin and DAB matrices of the software. On the DAB matrice, a threshold was adjusted for DAB detection according to intensity (grey values from 0 to 255) on representative stained versus not stained tissue areas. In a similar way, a threshold was also adjusted for tissue detection. These parameters were kept constant throughout the study for each immunostaining. Results were expressed as stained area (below threshold)/tissue area (below threshold) for β-tubulin IV and MUC5AC stainings and as percentage of positive cells (positive nuclei/total nuclei) for p63 and FOXJ1 staining. Ciliated cells (FOXJ1 and β-tubulin IV) were quantified outside of areas of obvious remodeling (i.e. with squamous metaplasia, Figure E1). Of note, involucrin immunostaining was only positive in 4 out of the 63 COPD patients (data not shown) and the inclusion of those regions did not change the quantification data presented in Figure 1.

For HBEC, 10 well-preserved areas of epithelium were taken for each patient for the quantification, using a Zeiss Axiovert 40 microscope and Axiovision software (Oberkochen, Germany). Quantification of the positive cells was manually done and data were expressed as the percentage of positive cells (positive cells or nuclei/total cells or nuclei).

Whole-mount staining

The membrane of the insert was cut and separated from its support before being washed twice with TBS with 0.1% Tween 20. Antigen retrieval was performed in citrate buffer containing 0.1% of triton X100 (Sigma) at pH 6.0 using cooker pressure treatment during 5 min at 15 psi.

The endogenous peroxidases were blocked by incubating the samples for 1h at RT with Bloxall reagent (Vector Laboratories Inc., Burlingame, CA) followed by blocking with normal goat serum (Abcam Inc., Cambridge, MA, USA) 5% with 0.3% H_2O_2 in TBS containing 0.1% Tween 20 during 2h at RT. The first primary antibody was anti-p63 (Dako; 1/2000). The inserts were then incubated overnight at 4°C with the primary antibody before being washed four times with TBS with 0.1% Tween 20. A corresponding poly-HRP secondary antibody (Thermo Fisher Scientific) was applied during 3h at RT. The insert were washed four times with TBS containing 0.1% Tween 20. HRP was then visualized by tyramide signal amplification using an AlexaFluor 488-conjugated tyramide (Thermo Fisher Scientific). After a new citrate buffer incubation step, the same procedure was applied consecutively with two different primary antibodies (anti- β-tubulin IV; Abcam; 0.17 µg/ml and anti-MUC5AC; Origene Herford; Germany; 0.2µg/ml) and 555 or 647 AlexaFluor-conjugated tyramide, respectively. After all, 3 sequential reactions with MUC5AC, beta-tubulin IV and p63 were performed. Finally, the nucleus nucleus was stained by incubation with Hoechst reagent (Thermo Fisher Scientific) and mounted with Dako fluorescence mounting medium (Dako, Carpinteria, CA, USA). Five optical sections for each stained inserts were acquired from the top to the bottom of the inserts by structured illumination using a Zeiss AxioImager equipped with an ApoTome.z1 module (20x Plan-Apochromat objective). From the acquired image stacks, maximum intensity projections were generated for analysis using the image analysis tool Author version 2017.2 (Visiopharm, Hørsholm, Denmark). P63, beta-tubulin IV and MUC5AC stained pixels were detected using a thresholding classification method. Results were expressed as percentage of stained area.

Western blot for epithelial markers and TGF-β signalling

Cells were lysed with 150µl of Laemmli's sample buffer containing 0.7M 2-mercaptoethanol (Sigma-Aldrich, Saint-Louis, USA) then kept at -20°C until proceeded. Samples were heated

at 100°C for 5 minutes, loaded in a SDS-PAGE gel and run at 100V for 15 minutes, then at 180V for 45 minutes. Cell proteins were transferred onto nitrocellulose membrane (Thermo Fisher, Waltham, USA) at 0.18A for 2 hours at RT. The membrane was blocked with 5% BSA (Sigma-Aldrich, Saint-Louis, USA) in TBS with 0.1% Tween 20 (Sigma-Aldrich, Saint-Louis, USA) for 1 hour at RT, washed and incubated with primary antibody overnight at 4°C. Membranes were then incubated for 1 hour at RT with secondary HRP-linked antibody. Prime chemiluminescent substrate was used to develop the immunochemical signal that was captured by a CCD-camera imager avoiding overexposure. Quantity One software was used for analysis. Each band individually selected was quantitated by densitometry and normalized for the corresponding GAPDH band intensity. Immunoreactive bands were revealed by chemiluminescence (GE Healthcare, Pittsburgh, USA) and detected by Chemidoc XRS apparatus (Bio-rad, Hercules, USA). Primary antibodies used were against human β-tubulin IV (clone ONS.1A6, Sigma-Aldrich, Saint-Louis, USA), human FOXJ1 (polyclonal, Sigma-Aldrich, Saint-Louis, USA), or human phospho-Smad2 (Ser465/467) / 3 (Ser 423/425) (Cell signaling, Danvers, USA), GAPDH (Sigma-Aldrich, Saint-Louis, USA). Secondary antibodies were peroxidase conjugated anti-mouse IgG (whole molecule) (Sigma-Aldrich, Saint-Louis, USA) and anti-rabbit IgG (Cell signaling, Danvers, USA).

RT-qPCR analysis for SPDEF, DNAI2, FOXJ1 messenger ribonucleic acid (mRNA)

Bronchial epithelial cells were lysed by RLT buffer (Qiagen, Hilden, Germany) containing 0.14M 2-mercaptoethanol (Sigma-Aldrich, Saint-Louis, USA) then kept at -80°C until proceeded. Total RNA was isolated from HBEC using the Rneasy® Plus Mini kit (Qiagen, Hilden, Germany). RNA (12µl out of 35µl of extracted RNA per HBEC sample) was reverse-transcribed with RevertAid H minus Reverse transcriptase kit (Thermo Fisher Scientific, Kent, United Kingdom) with 0.3 µg of random hexamer (Thermo Fisher Scientific, Kent, United Kingdom), 20U of RNase inhibitor (Thermo Fisher Scientific, Kent, United Kingdom)

and 1mM of each dNTP (Thermo Fisher Scientific, Kent, United Kingdom) following the manufacturer's protocol in a thermocycler (Applied Biosystems, Carlsbad, USA). The expression levels were quantified by real-time quantitative PCR with the iCycler IQ5 PCR (Bio-Rad, Hercule, USA). The reaction mix contained 2.5 µl of complementary desoxyribonucleic acid diluted 10-fold, 400nM of each primer (SPEDF Forward: GCA GCT AAC AGA CAC AGC; SPEDF Reverse: TTC TCC AAG CCT GTC CG; DNAI2 Forward: GCG ATT CAT ACA TCT GGG AC; DNAI2 Reverse: CAG CAG GCT ATC TGT CCA T; FOXJ1 Forward: CCT GGC AGA ATT CAA TCC G; FOXJ1 Reverse : GCG TAC TGG GGG TCA AT; RNA18S Forward: CGG CTA CCA CAT CCA AGG AA; RNA18S Reverse: ATA CGC TAT TGG AGC TGG AAT ACC; glyceraldehyde-3-phosphate deshydrogenase (GAPDH) Forward: ACC AGG TGG TCT CCT CTG AC; GAPDH Reverse: TGC TGT AGC CAA ATT CGT TG; ribosomal protein S18 (RPS18) Forward: TGT GGG CCG AAG ATA TGC T; RPS18 Reverse: TGA TCA CAC GTT CCA CCT CAT) and 2x iQTM SYBR® Green Supermix (Bio-Rad, Hercule, USA) in a final volume of 20 µl. The cycling conditions were 95°C for 3 minutes followed by 40 cycles of 95°C for 15s and 60°C for 30s. To control the specificity of the amplification products, a melting curve analysis was performed. Samples were run in duplicate and the copy number was calculated from the standard curve. Data analysis was performed using Bio-Rad iQ5 software (Bio-Rad, Hercule, USA). Expression levels of target genes were normalized to the geometric mean of the values for the 3 housekeeping genes.

Statistical analysis

Results were shown as scatter dot plots with medians and interquartile ranges. All tests used were non-parametric, Mann-Whitney U test (for unpaired data and the analysis of differences between 2 groups) and Friedman test (for paired data) followed by Dunns post hoc test (for multiple comparisons). Correlation coefficients were calculated using Spearman's rank

method. A p value less than 0.05 was considered as statistically significant. Statistical analyses were performed using IBM SPSS Statistics (version 24 for Windows, Chicago, USA) and figures were done using GraphPad Prism (version 7.00 for Windows; GraphPad Software, San Diego, USA; www.graphpad.com).

FOOTNOTES

Abbreviations:

ALI, air liquid interface

BSA, bovine serum albumin

COPD, chronic pulmonary obstructive disease

GOLD, global initiative for chronic obstructive lung disease

FEV1, forced expiratory volume in one second

FVC, forced vital capacity

HBEC, human bronchial epithelial cell

HRP, horseradish peroxidase conjugated

PBS, phosphate buffered saline

RT, room temperature

TBS, tris buffer saline

TGF-β, transforming growth factor-β

Table E1. Lactate deshydronase release in experiments.

LDH release in baso-lateral medium	Non treated (COPD)	TGF-β1 (2wks) (COPD)	Anti-TGF-β1 (2 wks) (COPD)	Mouse IgG (2 wks) (COPD)
**Optical density (340nm) at 0'**	1.137	1.145	1.147	1.144
**Optical density (340nm) at 5'**	1.134	1.144	1.146	1.144
**% LDH release**	**0.3**	**0.1**	**0.1**	**0**

LDH release in baso-lateral medium	Non treated (Non-smoker, n=2)	Non treated (COPD, n=7)
**Optical density (340nm) at 0'**	1.327<br>1.001	1.137<br>1.336<br>1.189<br>1.162<br>1.043<br>1.137<br>1.210
**Optical density (340nm) at 5'**	1.324<br>1.002	1.134<br>1.324<br>1.179<br>1.164<br>1.051<br>1.134<br>1.205
**% LDH release (mean)**	**0.2**	**0.3**

FIGURE LEGENDS

Figure E1: A - IHC pictures for MUC5AC (goblet cells) in large airways of 3 non-smoker, 3 smoker and 3 COPD patients. **B** - IHC pictures for ß-tubulin IV (ciliated cells) in large airways of 3 non-smoker, 3 smoker and 3 COPD patients. **C** - Quantification technique shown in a COPD patient (epithelium delimitation in red, tissue area in green, staining labelling in brown, exclusion of squamous metaplasia and goblet cells hyperplasia).

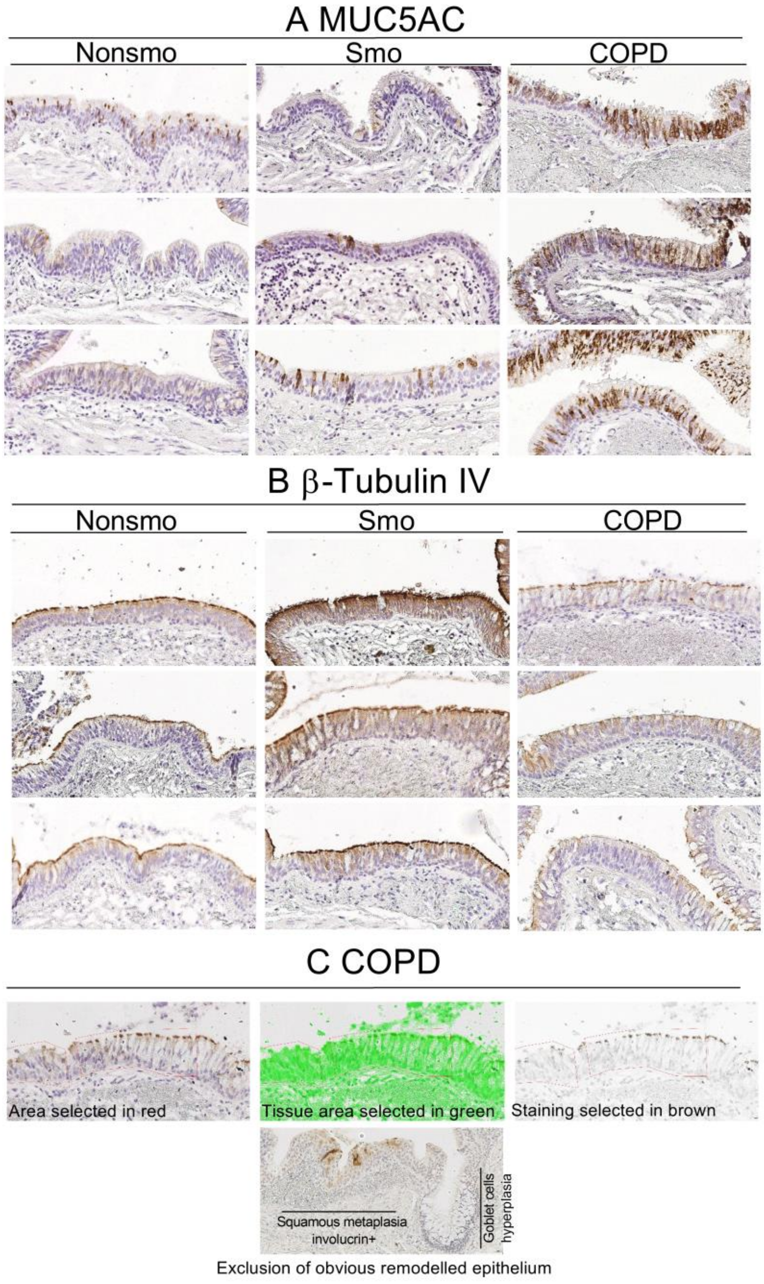

Figure E2: **A** - Quantification of MUC5AC staining in large airways expressed in percentage of positive area in non-smokers, former smokers and current smokers (n=63). **B** - Quantification of MUC5AC staining in small airways expressed in percentage of positive area in non-smokers, former smokers and current smokers (n=54). **C** - Correlation between MUC5AC staining in large airways and small airways (n=53). **D** - Correlation between MUC5AC staining in small airways and DL_{CO} in COPD patients (n=23).). Kruskal-Wallis test and Dunns's multiple comparisons test.

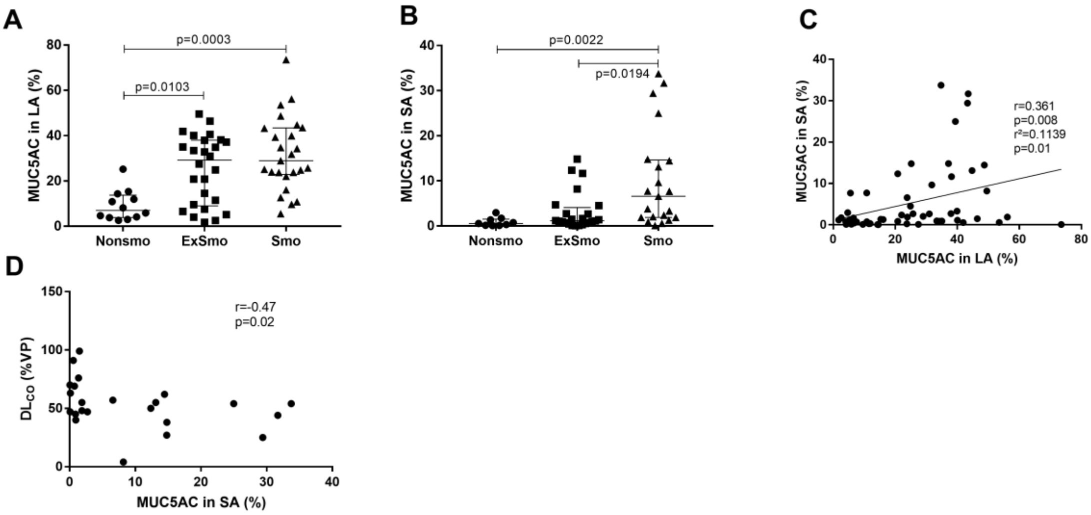

Figure E3: **A** - Correlation between MUC5AC staining in ALI-HBEC from COPD patients and FEV1 (n=19). **B** - Correlation between MUC5AC staining in ALI-HBEC from COPD patients and FEV1/FVC (n=19). **C** - Correlation between ß-tubulin IV staining in ALI-HBEC from current and former smokers and pack-years (n=30). **D** - Correlation between MUC5AC staining in large airways and in ALI-HBEC (n=12).

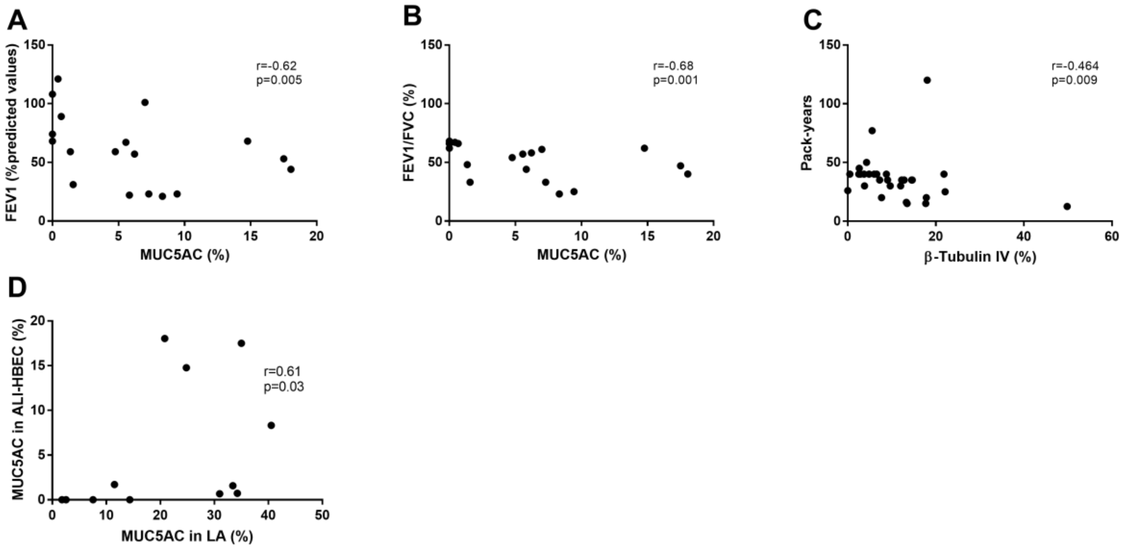

Figure E4: A - Correlation between DNAI2 mRNA/HK and pack-years in ALI-HBEC from former and current smokers (n=15).

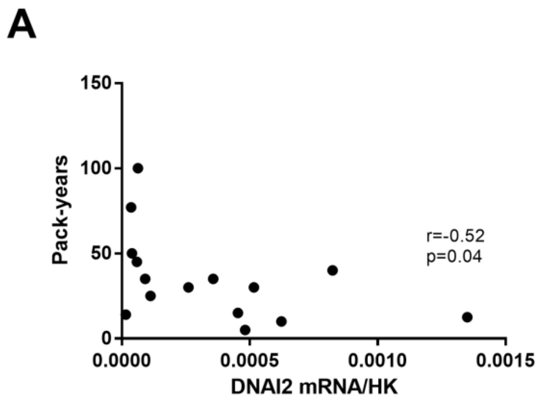

Altered generation of ciliated cells in chronic obstructive pulmonary disease

Authors: Sophie Gohy[1,2] MD PhD, François M. Carlier[1] MD, Chantal Fregimilicka[3], Bruno Detry[1], Marylène Lecocq[1], Maha Zohra Ladjemi[1] PhD, Stijn Verleden[4] PhD, Delphine Hoton MD[5], Birgit Weynand[6] MD, Caroline Bouzin[3] PhD and Charles Pilette[1,2] MD PhD.

Figure 3E – WB – FOXJ1 (50kDa)

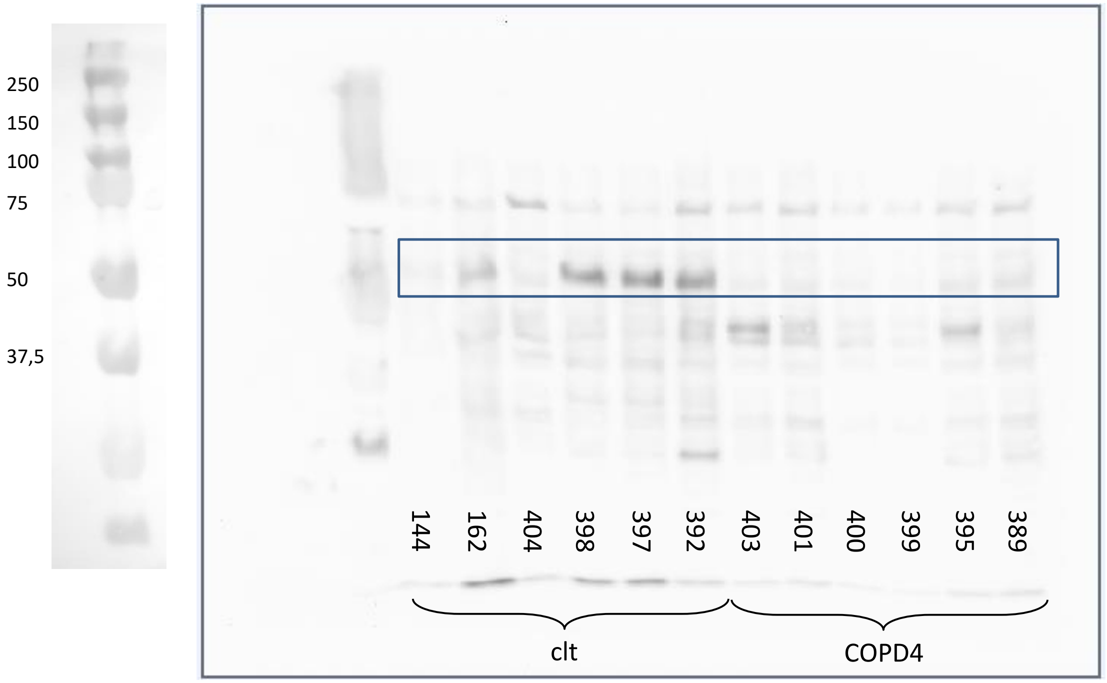

Figure 3E – WB – β-Tubulin IV (50kDa) and GAPDH (37kDa)

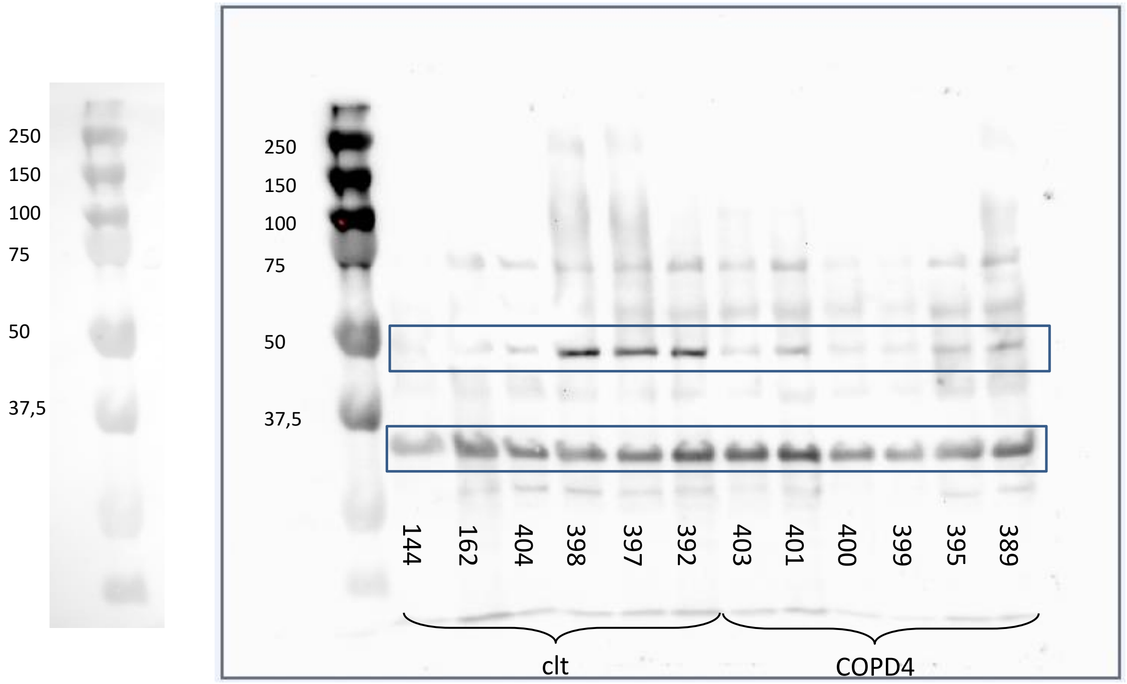

Figure 3E – WB – GAPDH (37kDa)

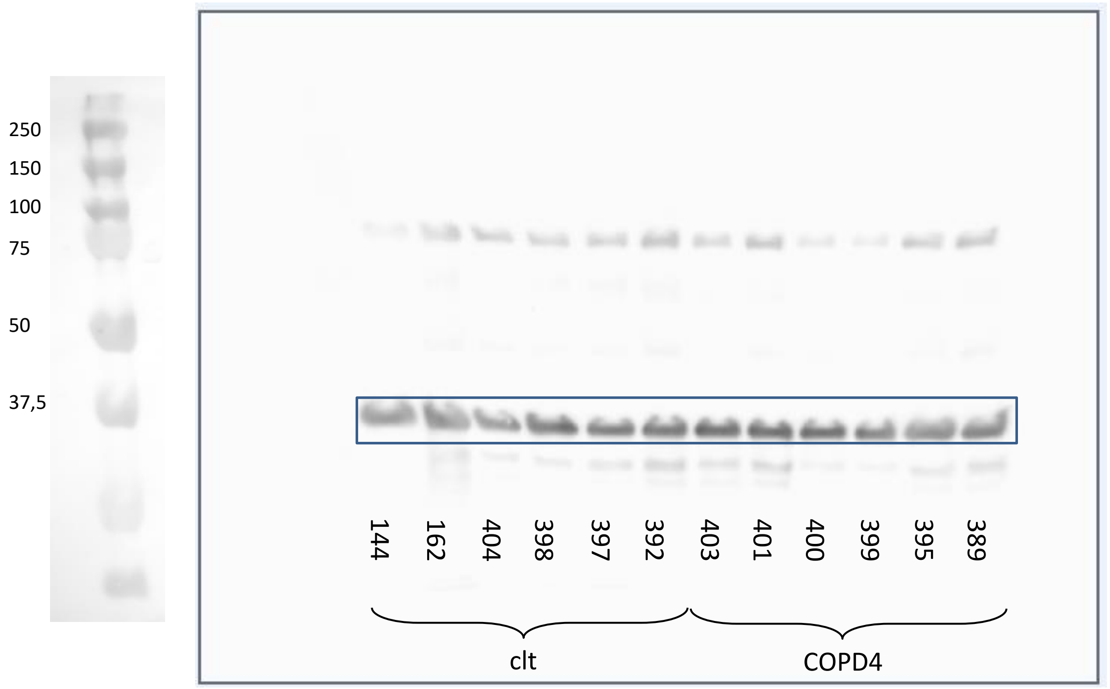

Figure 3E – WB – Molecular weight

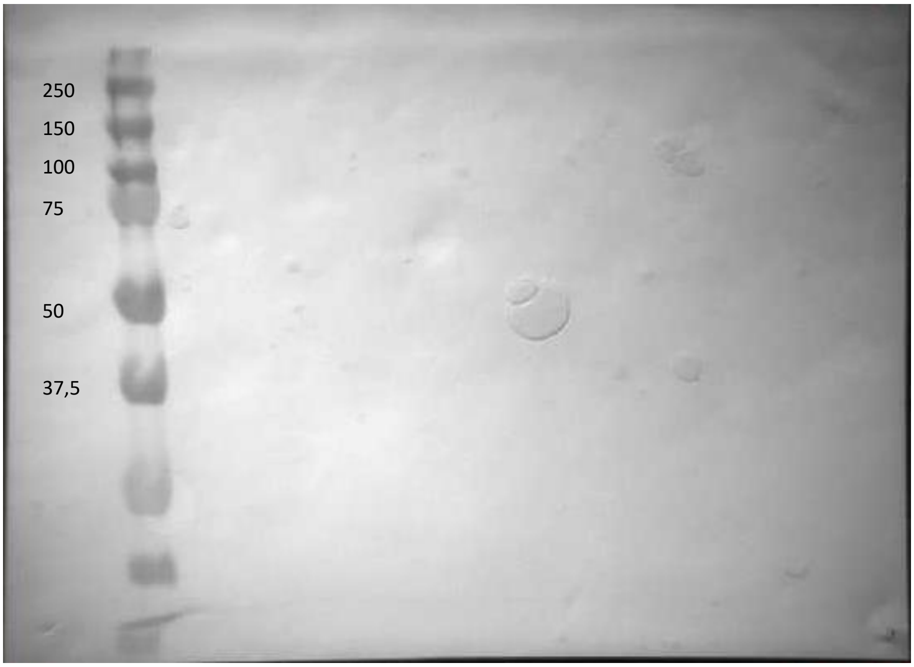

Figure 3E

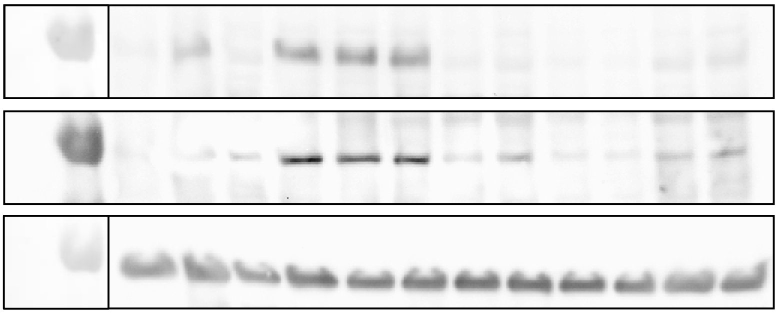

Figure 7E – WB – β-tubulin IV (50kDa)

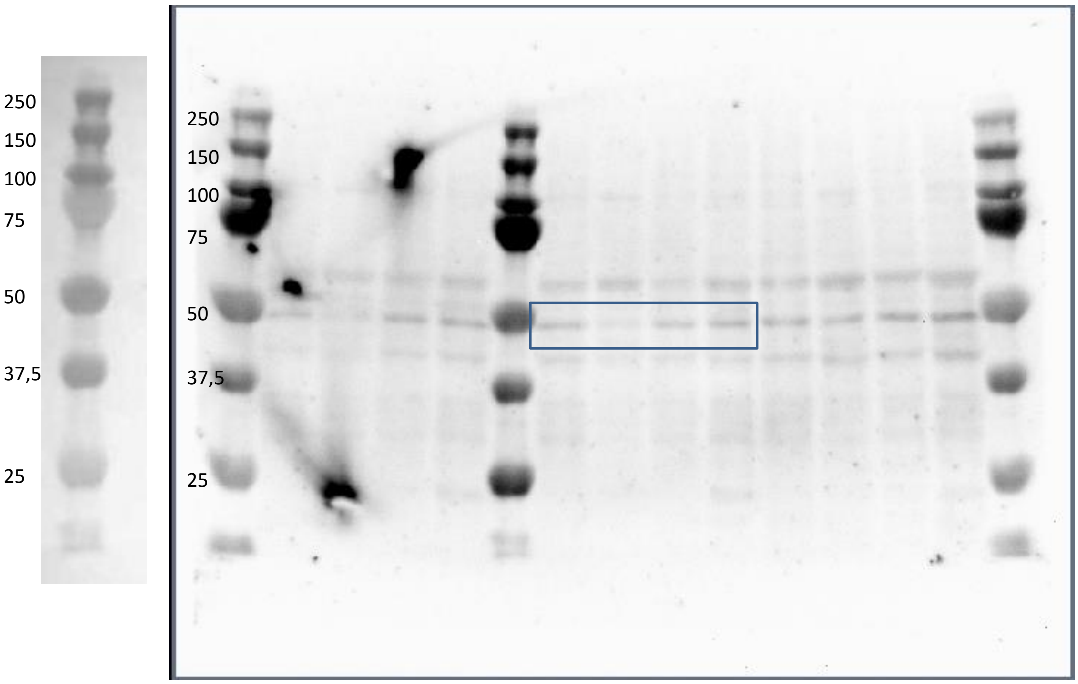

Figure 7E – WB – GAPDH (37kDa)

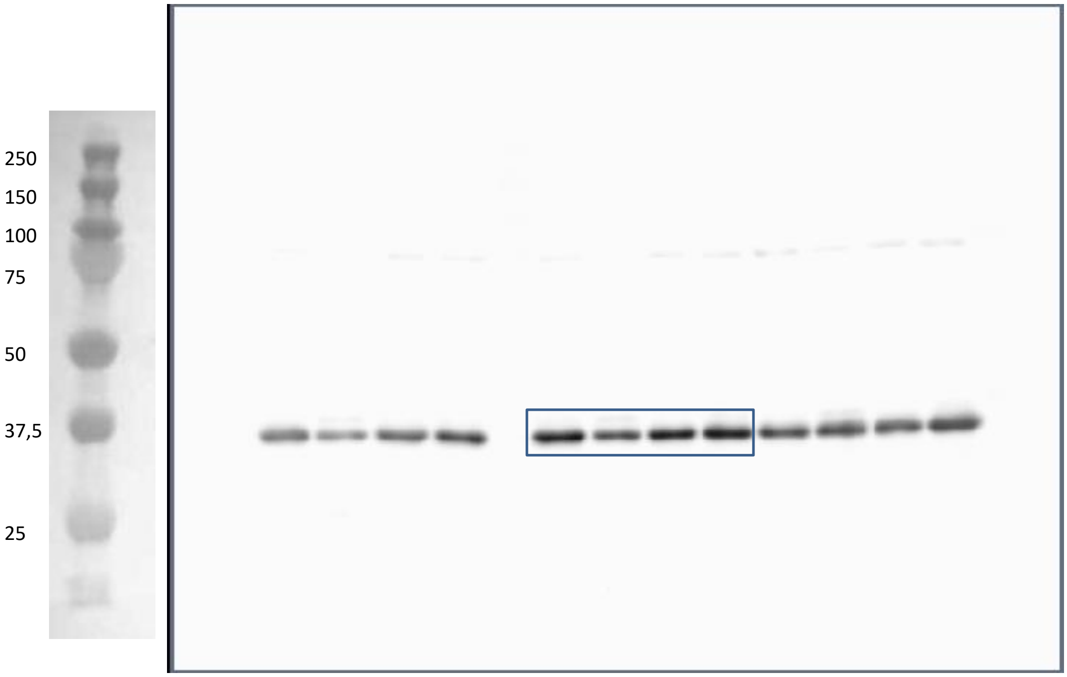

Figure 7E – WB – Molecular weight

250
150
100
75
50
37,5
25

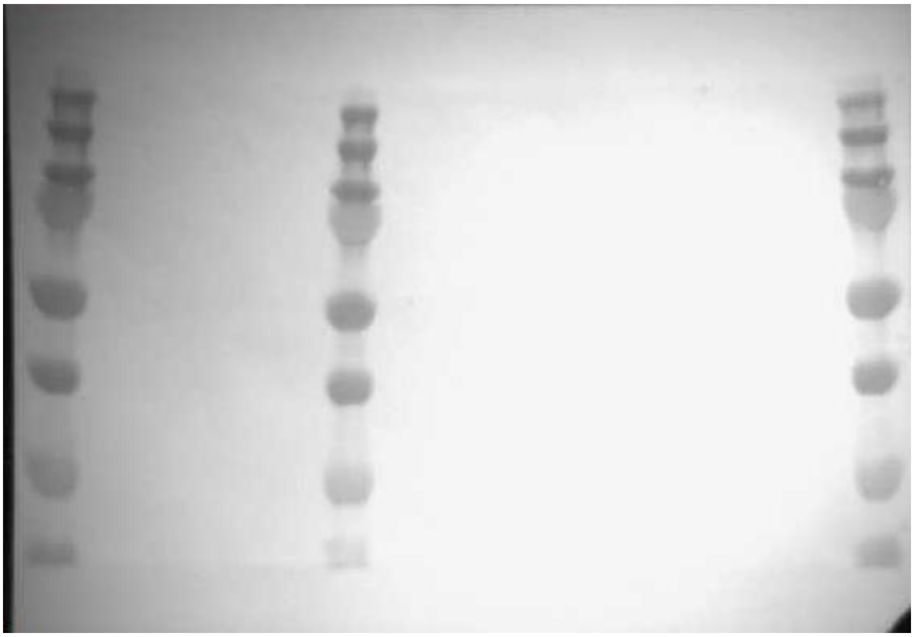

Figure 7E – WB – FOXJ1 (50kDa)

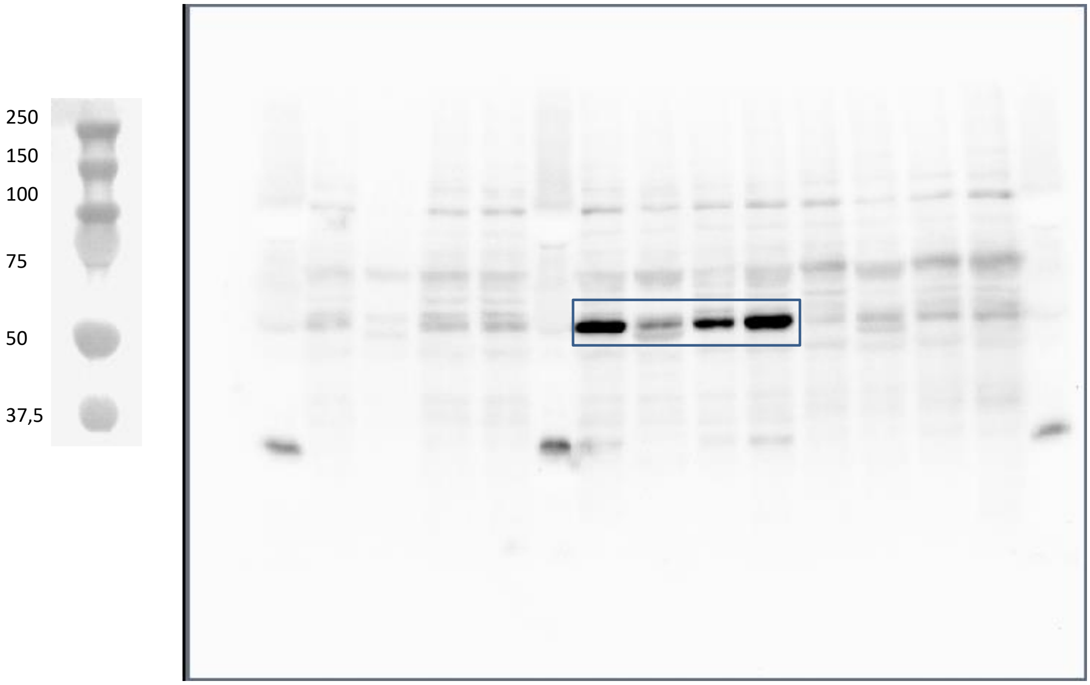

Figure 7E – WB – GAPDH (37kDa)

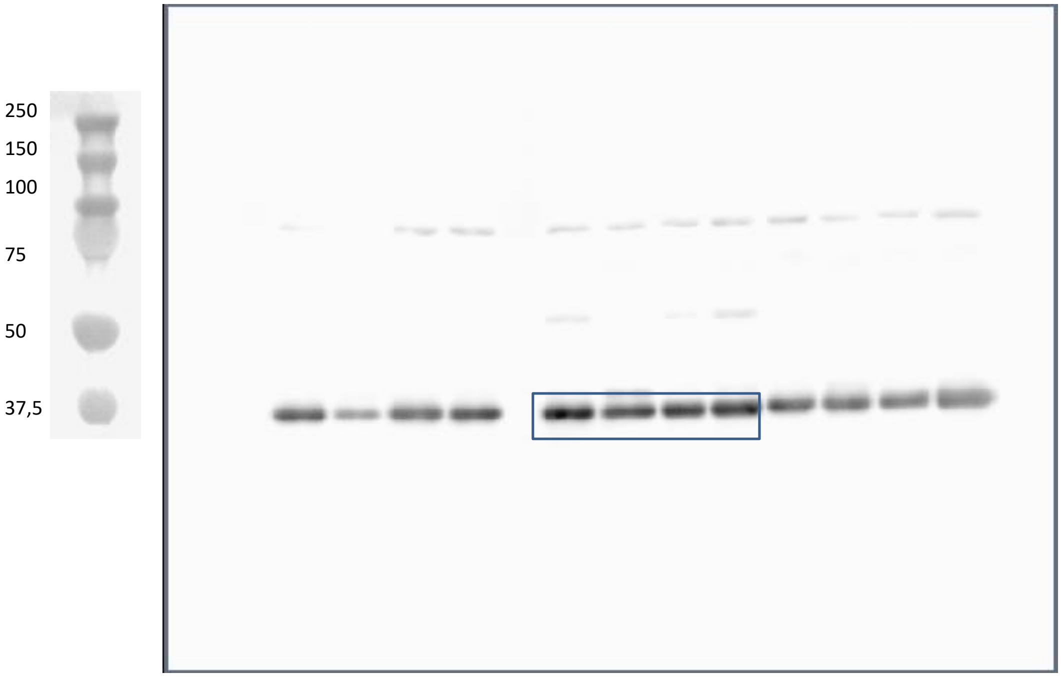

Figure 7E – WB – Molecular weight

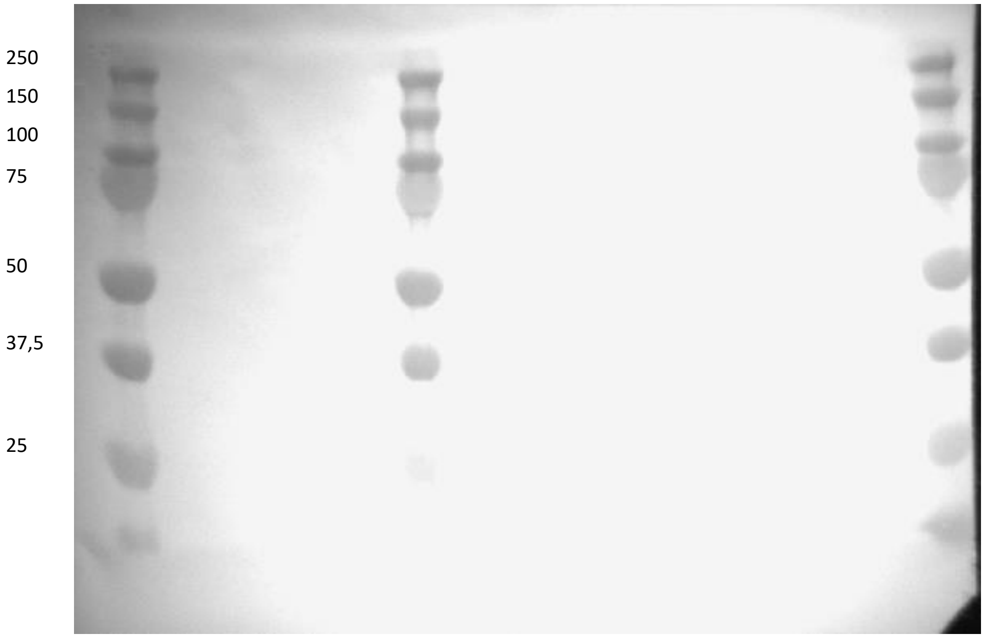

Figure 7E – WB – Psmad2/3 (68kDa)

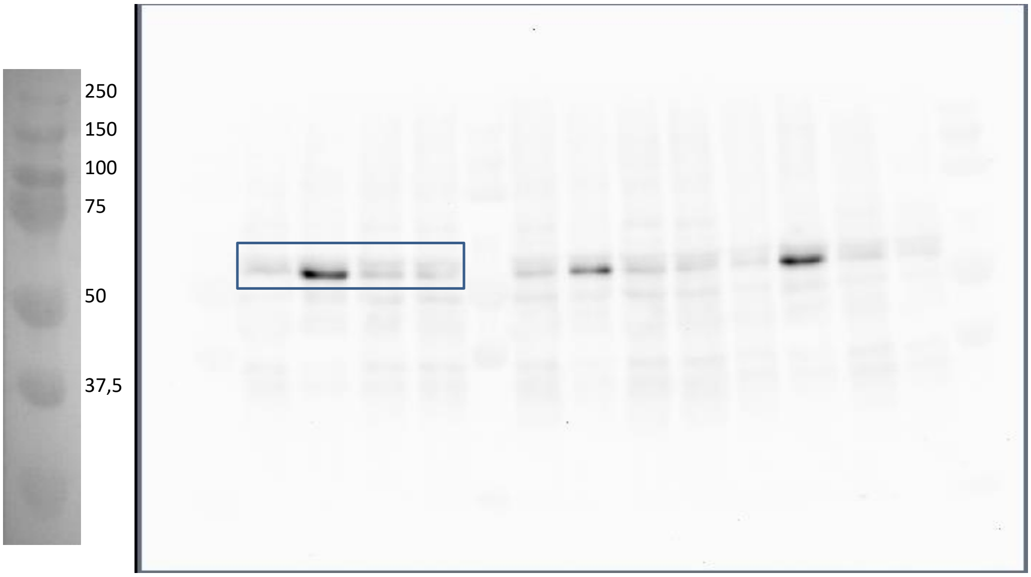

Figure 7E – WB – GAPDH (37kDa)

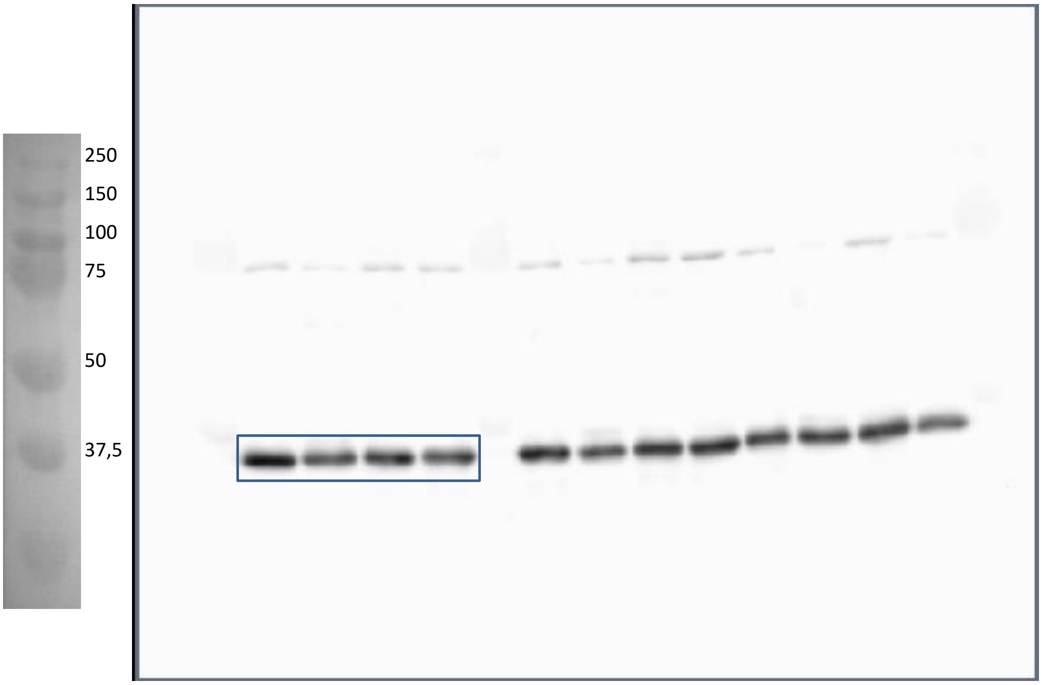

Figure 7E – WB – Molecular weigth

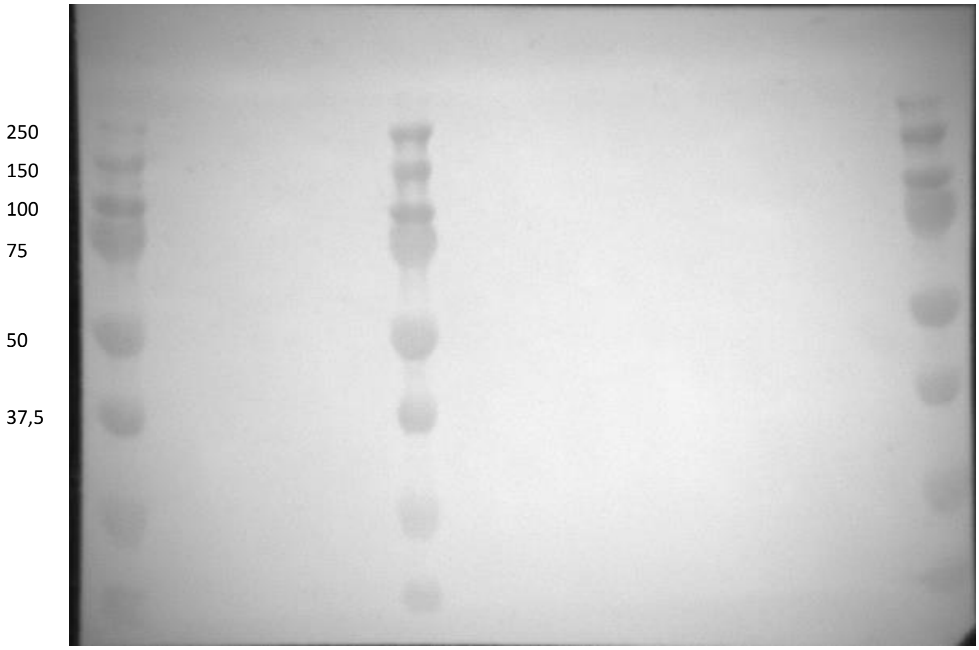

Figure 7E

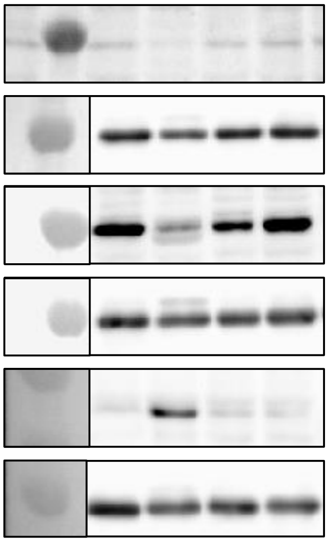

Supplement: Supplementary file 1 — Supplementary data [file 41598_2019_54292_MOESM1_ESM.pdf]
